# MeltMan: Optimization, Evaluation, and Universal Application of a qPCR System Integrating the TaqMan qPCR and Melting Analysis into a Single Assay

**DOI:** 10.1371/journal.pone.0151204

**Published:** 2016-03-31

**Authors:** Alexander Nagy, Lenka Černíková, Eliška Vitásková, Vlastimil Křivda, Ádám Dán, Zuzana Dirbáková, Helena Jiřincová, Bohumír Procházka, Kamil Sedlák, Martina Havlíčková

**Affiliations:** 1 Laboratory of Molecular Methods, State Veterinary Institute Prague, Prague, Czech Republic; 2 National Food Chain Safety Office, Veterinary Diagnostic Directorate, Molecular Biology Laboratory, Budapest, Hungary; 3 Department of Virology, State Veterinary Institute Zvolen, Zvolen, Slovak Republic; 4 National Reference Laboratory for Influenza, National Institute of Public Health, Prague, Czech Republic; 5 Department of Informatics and Biostatistics, National Institute of Public Health, Prague, Czech Republic; 6 Department of Virology and Serology, State Veterinary Institute Prague, Prague, Czech Republic; Chang Gung University, TAIWAN

## Abstract

In the present work, we optimised and evaluated a qPCR system integrating 6-FAM (6-carboxyfluorescein)-labelled TaqMan probes and melting analysis using the SYTO 82 (S82) DNA binding dye in a single reaction. We investigated the influence of the S82 on various TaqMan and melting analysis parameters and defined its optimal concentration. In the next step, the method was evaluated in 36 different TaqMan assays with a total of 729 paired reactions using various DNA and RNA templates, including field specimens. In addition, the melting profiles of interest were correlated with the electrophoretic patterns. We proved that the S82 is fully compatible with the FAM-TaqMan system. Further, the advantages of this approach in routine diagnostic TaqMan qPCR were illustrated with practical examples. These included solving problems with flat or other atypical amplification curves or even false negativity as a result of probe binding failure. Our data clearly show that the integration of the TaqMan qPCR and melting analysis into a single assay provides an additional control option as well as the opportunity to perform more complex analyses, get more data from the reactions, and obtain analysis results with higher confidence.

## Introduction

Currently, the polymerase chain reaction (PCR) is the most ubiquitous molecular biology tool in use. Since its invention in 1983 [1], the method has undergone substantial improvements in terms of chemistry, instrumentation, and consumables which has led to the development of a brand new industrial segment in the. Nowadays the PCR technology represents an important portfolio in the life science industry.

The PCR is a copy technique enabling to multiply a nucleic acid (NA) sequence of interest on the basis of a pre-existing template. The copies, referred to as amplicons, are then visualised after the end of the reaction (in conventional or endpoint PCR) or during the amplification. The later approach enabling to monitor the amplification in real time is allowed by the introduction of fluorescence labelling into the assay, which represents one of the greatest improvements of the technique In addition, it has also the capacity to measure the starting nucleic acid template amounts. Therefore, the assay is termed fluorescence-based real-time quantitative PCR (qPCR or RT-qPCR) [2].

Early after the implementation of the fluorescence the qPCR techniques diverged into two categories depending on the fluorescence option used. The first group utilizes DNA-associating dyes and enables amplicon detection in a sequence-non-specific manner while the second one relies on sequence-specific detection and employs fluorogenic oligoprobes or primers [3]. The DNA-associating dyes allow constant monitoring of amplification through direct interaction with DNA usually by intercalation. Since this interaction is sequence-non-specific, subsequent melting analysis is required for amplicon discrimination which is based on differences in temperature-dependent dissociation of double-stranded DNA.

The sequence specific qPCR has a plethora of various modifications. Of them, the hydrolysis probe technique [4], later also called “TaqMan”, is the most used. The TaqMan qPCR relies on a pair of primers and a dually labelled oligoprobe. During the reaction, the signal is emitted only after the probe hydrolysis. This requires a unique arrangement of three target sequence-specific oligonucleotides. The hydrolysis probe, therefore, introduces an additional level of specificity into the qPCR reaction. Moreover, several differently labelled probes can be utilized in the same assay allowing real time multiplexing. These properties along with the simplicity and a relatively easy assay design make the TaqMan the most popular qPCR technique. Especially in diagnostic microbiology, the TaqMan qPCR represents one of the most powerful diagnostic method which is frequently used as a first screening tool for many important pathogens.

Given the three-oligonucleotides, the TaqMan qPCR results inherently suggest high level of confidence. But how reliable really is the technique? Do the TaqMan amplification curves always mean positivity? Is there always a correspondence between the amplification curve and a specific amplicon? Conversely, does the absence of the specific signal always mean true negativity?

Paradoxically, the fluorescent probe may also contribute substantially to drawbacks of the TaqMan qPCR, mainly by increasing the susceptibility of the assay towards the template mutations. Mutations in the probe binding region may decrease the probe binding or completely abolish it (Profiles 1A-D in S1 File) thus leading to miscellaneous results or false negativity. This phenomenon is especially characteristic for highly variable RNA viruses [5–9] and represents a significant issue in diagnostic microbiology. In addition, late reaction curves referred to as “tails”, and further zig-zag, sigmoidal, or other weird shaped curves may be sporadically generated during the reaction (Profiles 2A, and 3 in S1 File). Such profiles can be easily misinterpreted or, when it occurs in the negative control, may indicate contamination. However, additional steps are required to clarify the results obtained. How to limit these undesirable effects?

Recent works comparing different DNA binding dyes in qPCR have shown superior properties of certain SYTO dye family members which outperform the conventionally used Sybr Green (SG; [10, 11]). Unlike SG, the SYTO dyes have no sequence binding preference and neither inhibit the amplification nor influence the melting temperature even at higher concentrations. One of them, SYTO 82 (further referred to as S82) exhibits outstanding PCR reaction compatibility [10, 11]. S82 is an orange fluorescent nucleic acid stain allowing continuous amplification monitoring and subsequent melting analysis in the yellow channel. This property provides the opportunity to integrate the sequence specific TaqMan qPCR using FAM labelled probes with a sequence-non-specific detection in a single assay. Such S82-TaqMan qPCR or “MeltMan” system would increase the reliability of the analysis with great potential to minimize the aforementioned drawbacks of the TaqMan technique.

In the present work, we characterized the effects of the S82 dye on the TaqMan qPCR reaction utilizing FAM labelled probes. We investigated how different S82 concentrations affect the basic TaqMan reaction parameters including the FAM fluorescence and sensitivity of the melting analysis. In the next step, we attempted to define the optimal S82 concentration in the TaqMan reaction. Finally, the applicability of the MeltMan concept in routine use was investigated through various qPCR and RT-qPCR assays in a joint effort of four PCR diagnostic laboratories. The specific problems regarding to routine MeltMan qPCR applications were also highlighted.

## Materials and Methods

### Specimens and nucleic acid standards

The qPCR and RT-qPCR assays for viral or bacterial detection were tested on various field specimens including simple (intestines, lungs) or pooled organ suspensions, blood, serum, faeces, semen, swabs (rectal, ocular, nasopharyngeal, buccal, or epidermal) and pure viral and bacterial cultures. The specimens originated from various animal species including dogs, cats, cows, pigs, horses, goats, sheep, chickens, and parrots. The specimens for qPCR assays designed for meat species identification and monitoring of food adulterations were food and dairy products (lasagne, hamburgers, beef, precooked or fried meat as well as milk, cheese, and yogurt). All the specimens were selected from our repository and were collected for diagnostic and not for research purposes. The ethical standards in animal welfare and protection are subject to inspection by the State Veterinary Administration of the Czech Republic

Nucleic acid (NA) standards for the Foot and Mouth Disease Virus (FMDV, [12]) and Influenza A Virus (IAV, [7]) assays (Table 1) at a concentration of 4nM were ordered from Integrated DNA Technologies.

**Table 1:** Amplicon lengths, thermoprofiles and probe modifications of the TaqMan qPCR and RT-qPCR assays included in the study.

TaqMan assay	Abbr.	Target gene	NA	Amplicon length (bp)	Amplification thermoprofile^a^			Probe modifications	Reference
					1	2	3		
Canine Parvovirus	CPV	VP2	ssDNA	93	95°C 30s	60°C 1min		FAM-MGB-NFQ	[40]
Feline Parvovirus	FPV			83					
Ovine DNA	n.a	Prolactin receptor	dsDNA	88	95°C 20s	62°C 30s	-	BHQ1-FAM	[41]
Equine DNA	n.a	Mt DNA	dsDNA	87	95°C 15s	50°C 1min	-	BHQ1-FAM	[42]
Caprine DNA	n.a	CytB	dsDNA	140	95°C 5s	62°C 1min	72°C 1min	BHQ1-FAM	[17]
Canine DNA	n.a	MC1R	dsDNA	72	95°C 15s	60°C 1min	-	FAM-MGB-NFQ	[43]
Bovine DNA	n.a	Beta-actin	dsDNA	96	95°C 20s	62°C 30s	-	BHQ1-FAM	[41]
African Swine Fever Virus	ASFV	VP72	dsDNA	257	95°C 10s	58°C 30s	-	BHQ1-FAM	[44]
				68	95°C 10s	60°C 30s	-	FAM-NFQ, LNA^d^	[45]^c^
Swine DNA	n.a	Beta-actin	dsDNA	103	95°C 20s	62°C 30s	-	BHQ1-FAM	[41]
Ovine Herpesvirus 2	OvHv-2	Tegument protein	dsDNA	147	95°C 15s	60°C 1min	-	BHQ1-FAM	[15]
Burkholderia mallei	n.a	flip	dsDNA	121	95°C 25s	63°C 1min	-	BHQ1-FAM	[46]
Burkholderia pseudomallei	n.a	ORF1	dsDNA	110	95°C 10s	60°C 45s	-	BHQ1-FAM	[47]
Infectious Bovine Rhinotracheitis	IBR	gpB	dsDNA	97	95°C15s	60°C 45s	-	BHQ1-FAM	[48]
Parapoxviruses	n.a	B2L env. prot.	dsDNA	95	95°C 10s	55°C 10s	72°C 10s	FAM-MGB-NFQ	[49]
Koi herpesvirus	KHV	ORF89	dsDNA	78	95°C 15s	60°C 1min	-	BHQ1-FAM	[50]
Chlamydia sp.	n.a	23S rDNA	dsDNA	129	95°C 15s	59°C 1min	-	BHQ1-FAM	[51]
Equine Herpesvirus 1^c^	EHV1	gpB	dsDNA	63	95°C 15s	64°C 1min	-	FAM-MGB-NFQ	[14]
Equine Herpesvirus 4	EHV4			60					
Psittacine circovirus	PBFDV	ORF1	ssDNA	212	95°C 15s	60°C 30s	72°C 30s	BHQ1-FAM	Černíková et al. (unpublished)
Celery DNA^c^	n.a	Mtd	dsDNA	101	95°C 15s	60°C 1min	-	BHQ1-FAM	[16]
Chicken DNA^c^	n.a	TGF-BETA3	ssDNA	76	95°C 10s	60°C 1min	-	BHQ1-FAM	[17]
Mycoplasma sp.^c^	n.a	Tuf	dsDNA	147	95°C 10s	59°C 35s	72°C 10s	FAM-BHQ, LNA	[52]
Bovine Viral Diarrhoea Virus 1	BVDV1	5’UTR	ssRNA	168	95°C 10s	60°C 1min	-	BHQ1-FAM	[53]
Foot and Mouth Disease Virus	FMDV	5’UTR	ssRNA	120	95°C 15s	60°C 1min	-	BHQ1-FAM	[12]
	FMDV-2	3D	ssRNA	107	95°C 15s	60°C 1min	-	BHQ1-FAM	[54]
Tick-borne Encephalitis Virus	TBEV	NS1	ssRNA	98	95°C 15s	60°C 40s	-	FAM-MGB-NFQ^d^	[18]
Influenza A Virus	IAV	M segment	ssRNA	182	95°C 10s	60°C 20s	72°C 10s	FAM-NFQ, LNA	[7]
Pandemic Influenza Virus H1	H1N1pdm	HA	ssRNA	116	95°C 15s	55°C 30s	-	BHQ1-FAM	[55]
Vesicular Stomatitis Virus	VSIV	L gene	ssRNA	266	95°C 15s	54°C 30s	72°C 1min	FAM-MGB-NFQ	[56]
	VSNJV-1			227					
	VSNJV-2							BHQ1-FAM	
Bovine Respiratory Syncytial Virus	BRSV	F gene	ssRNA	85	95°C 30s	50°C 30s	72°C 45s	BHQ1-FAM	[57]
Equine Arteritis Virus	EAV	ORF7	ssRNA	204	95°C 15s	60°C 1min	-	BHQ1-FAM	[19]
Swine Vesicular Disease Virus	SVDV-25	5’UTR-IRES	ssRNA	82	95°C 15s	60°C 1min	-	BHQ-FAM	[58]
	SVDV-3			68					
Avian Influenza Virus	AIV	M segment	ssRNA	101	95°C 10s	60°C 20s	-	BHQ1-FAM	[32]^b^
Avian Influenza Virus H5	AIV-H5	H5 segment HA2	ssRNA	152	95°C 10s	54°C 30s	72°C 10s	BHQ1-FAM	
Avian Influenza Virus H5	AIV-H7	H7 segment HA2	ssRNA	133	95°C 10s	54°C 30s	72°C 10s	BHQ1-FAM	[59]
Avian Influenza Virus H9	AIV-H9	H9 segment HA2	ssRNA	69	95°C 45s	54°C 45s	-	BHQ1-FAM	[33]
Avian Influenza Virus N1	AIV-N1	N1 segment	ssRNA	131	95°C 15s	55°C 30s	72°C 40s	BHQ1-FAM	[60]
Infectious Bronchitis Virus^c^	IBV	5’UTR	ssRNA	143	95°C 15s	60°C 1min	-	BHQ1-FAM	[61]

^a^ The initial denaturation and reverse transcription thermoprofiles were listed in point 2.4. Each thermoprofile was repeated 45 times.

^b^ The IAV-H5 primers were modified by the Avian Influenza EU Reference Laboratory, Animal and Plant Health Agency, UK.

^c^ These assays were not included in the statistical analysis.

^d^ UPL- Universal Probe Library.

### Nucleic acid extraction

Total NA was extracted using the MagNAPure Compact (Total NA Extraction Kit I), MagNAPure LC (Total NA Extraction Kit), and MagNAPure 96 (DNA and Viral NA Small Volume Kit; all from Roche) extractors, with input sample volumes from 100 to 400μl and elution volumes of 50 or 100μl, respectively.

### TaqMan qPCR assays and reaction mix compositions

The TaqMan assays and probe modifications used for the evaluation of the MeltMan system are summarized in Table 1. All the primers and probes used were obtained from Generi-Biotech, Czech Republic, except the minor groove binder (MGB) and locked nucleotide (LNA) modified probes, with the former supplied by Life Technologies and the latter by Roche or Integrated DNA Technologies. Each probe was labelled with a 6-FAM reporter dye and a corresponding quencher.

All of the reaction mixes were prepared on the basis of the QuantiTect Probe PCR or QuantiTect Probe RT-PCR Kits (Qiagen), unless stated otherwise. The reactions were set up in a final volume of 25μl (20μl reaction mix and 5μl of NA extract) by using white opaque and foil-sealed plates (LC 480 multiwell plate 96, Roche) or white opaque thin-walled and flat-cap sealed eight-tube strips (Bioplastics). Each of the particular TaqMan assays was set up in two subsets. The first one, called the standard or no S82 subset, contained 0.6μM of primers and 0.2μM of hydrolysis probe. The second one, referred to as the test or S82 subset, contained 0.6μM of primers, 0.4μM of probe and S82 dye (Life Technologies) in a final concentration of 0.8μM. The working solutions of S82 were prepared by dilution in nuclease-free water (Qiagen) and were stored at -20°C with freezing/thaw no more than2-3 times.

### Amplification and melting analysis thermoprofiles

The above-mentioned subsets were analysed on the CFX96 (BioRad) thermal cycler, unless stated otherwise. For the QuantiTect Probe PCR Kit, the thermoprofiles universally started with an initial activation at 95°C for 15 min and for the QuantiTect Probe RT-PCR Kit, with a reverse transcription at 50°C for 30 min and 95°C for 15 min. These initial steps were followed by 45 cycles of an assay-specific thermoprofile (Table 1) with a signal acquisition in the FAM and VIC channels at the end of the hybridization (for three-step thermoprofiles) or annealing/extension phase (for two-step thermoprofiles). The amplification was immediately followed by melting analysis ramping from 50°C to 95°C in 0.5°C increments, plate read for 0.5s, and signal acquisition in the VIC channel. For selected reactions the melting analysis was repeated in the LC480 v.1 thermal cycler (Roche) with initial pre-incubation at 40°C for 2 min and a temperature ramp at a rate of 0.06°C/s to 95°C with a continuous signal acquisition mode (10 counts/°C). To read the S82 emission in the LC480v.1 instrument, the following detection format was implemented: excitation and emission filters of 523 and 568, a melt factor of 1.2, a quant factor of 20, and maximum integration time of 1s.

The Cq values were estimated by analyzing the S82 and no S82 data as a single pool using the automatic threshold and baseline cycles option of the CFX Manager Software v3.1. For point 3.7, three additional approaches were implemented i) automatic data analysis separately for the S82 and no S82 reaction subsets, employing the automatic threshold and baseline cycles option; ii) manual threshold adjustment separately for each subsets, and iii) direct Cq inference from the FAM curve trajectories using the qPCR Miner [13] web-based application.

The Cq values were evaluated by constructing the Cq plot and ΔCq scatter plot separately for each of the four baseline approaches used. Within the Cq plot, the no S82 subset Cq values were plotted against the S82 subset values. The ΔCq was calculated according to the formula ΔCq = Cq[no S82]-Cq[S82].

### SYTO 82 dilution gradient

Two TaqMan qPCR assay reaction mixes, IAV and FMDV, (Table 1) with 0.6 μM of primers, 0.2 μM of probe, and S82 ranging from 0 to 10μM/reaction were prepared as qPCR. The mixes were then used to amplify the respective synthetic DNA standards with a fixed concentration of 1e4 copies/μl in three replicates including a non-template control (NTC). The reactions were run under the conditions and thermoprofiles according to Table 1 and points 2.3 and 2.4.

### Standard curve analysis

Standard curve analysis was performed on the basis of the FMDV and IAV TaqMan qPCR assays. Both of the assays were prepared in two subsets (point 2.3) and were used to amplify a 10-fold dilution gradient of the corresponding synthetic DNA standard, covering a quantitative range of five magnitudes (from 1e2 to 1e6), in triplicates including NTCs. Both of the reaction subsets were analysed in a single run according to the thermoprofile in Table 1 and point 2.4.

### Relative fluorescence signal evaluation

The relative fluorescence unit (RFU) values of the amplification curves gathered in the FAM and VIC channels and the derivative melting curve RFU values (-d(RFU)/dT) obtained in the VIC channel were analysed from the raw CFX96 run data.

In an effort to compare the amplification curve fluorescence between the no S82 and S82 reactions, we defined two parameters: the d(RFU) and the steepness *k*_._ The d(RFU) is the fluorescence value at a specific point of the sigmoidal trajectory which corresponds with its first derivative maximum. Then, the d(RFU) expressed as a percentage reflects the differences of the S82 relative to the no S82 reactions. The steepness was defined as the slope of the linear regression line driven along the exponential region of the amplification curve. The exponential region was determined from those RFU data points which gave the highest correlation (R^2^ >0.995) of the regression line.

To characterise the melting peaks, we introduced the peak height H and peak proportionality. The peak height expresses the background-to-tip ratio in -d(RFU)/dT values where the background was inferred from the melting peak’s left-tail local minimum. The melting peak proportionality means the peak height expressed as percentage.

### Analysis of the reaction sensitivity

Four TaqMan qPCR, Equine Herpesvirus 1 (EHV-1, [14]), Ovine Herpesvirus 2 (OvHV-1, [15]), Celery DNA [16], and Chicken DNA [17], and three RT-qPCR assays, Tick-Borne Encephalitis Virus (TBEV, [18]), IAV [7], and Equine Arteritis Virus (EAV, [19]) were prepared in two reaction subsets: no S82 and S82 where the later contained 0.4 μM of the probe and 0.8μM of S82/reaction (Table 1). As the templates, nuclease-free water-diluted positive field specimens were investigated to obtain Cq values ≥30 were investigated. Each template was analysed in ten replicates per subset including one NTC. The amplification and melting analysis were carried out according to points 2.3, 2.4, and Table 1.

### Electrophoresis

Electrophoretic analyses were performed on the TapeStation 2000 instrument (D1000 Screen Tape and D1000 Reagents kits; all from Agilent Technologies) or in horizontal electrophoresis with 2% agarose gel (TAE buffer, and 10V/cm).

### Sequence analysis

The amplicons of interest were purified directly or from the agarose gel (High Pure PCR Purification Kit; Roche). Sequencing reactions were prepared using the Big Dye Terminator Cycle Sequencing Kit v3.1 and evaluated on 3130 or 3500 Genetic Analysers (all from Life Technologies).

### Statistical analysis

The Cq and d(RFU) FAM values of the chapter 3.7 data were statistically analysed based on the two sample Student t-test using the IBM-SPSSv.23 software. The statistical evaluation was performed separately for the DNA (N = 430) and RNA (N = 299) data pools where the mean values and the paired S82 and no S82 subset statistics were determined separately for each assay and each baseline strategy (N = from 8 to 55). All the results were evaluated at a significance level of 5%, and the mean, standard deviation, and p values were provided.

## Results and Discussion

### Spectral characterization of the SYTO 82 dye

S82 belongs to the group of SYTO orange dyes which forms, along with the green, blue, and red groups, a broad family of cell-permeant nucleic acid stains. From the structural point of view, they are substituted unsymmetrical cyanine dyes derived from thiazole orange as the progenitor [20]. The exact chemical structure of the S82 dye and the DNA binding mechanism are not known. Nevertheless, the entire SYTO family is characterized by extremely low intrinsic fluorescence (quantum yields <0.01) in the free state. However, NA binding and subsequent excitation results in bright fluorescence emission (quantum yields typically of> 0.4; [21]). The absorption and fluorescence emission maxima of S82 are λabs = 541 and λem = 560 nm (Fig A in S2 File) which enables signal gathering in the yellow channel.

### Influence of the SYTO 82 dye on the TaqMan assay

The effect of the S82 dye on the TaqMan reaction was tested in a concentration gradient from 0 to 10μM of S82 per reaction using the IAV and FMDV assays (Table 1) which amplified a synthetic DNA template in a fixed concentration of 1e4 copies/μl. All dilution points were performed in triplicates including an NTC. We assessed the effects of S82 on the FAM signal, Cq values, and reaction efficiency.

**Fig 1:**
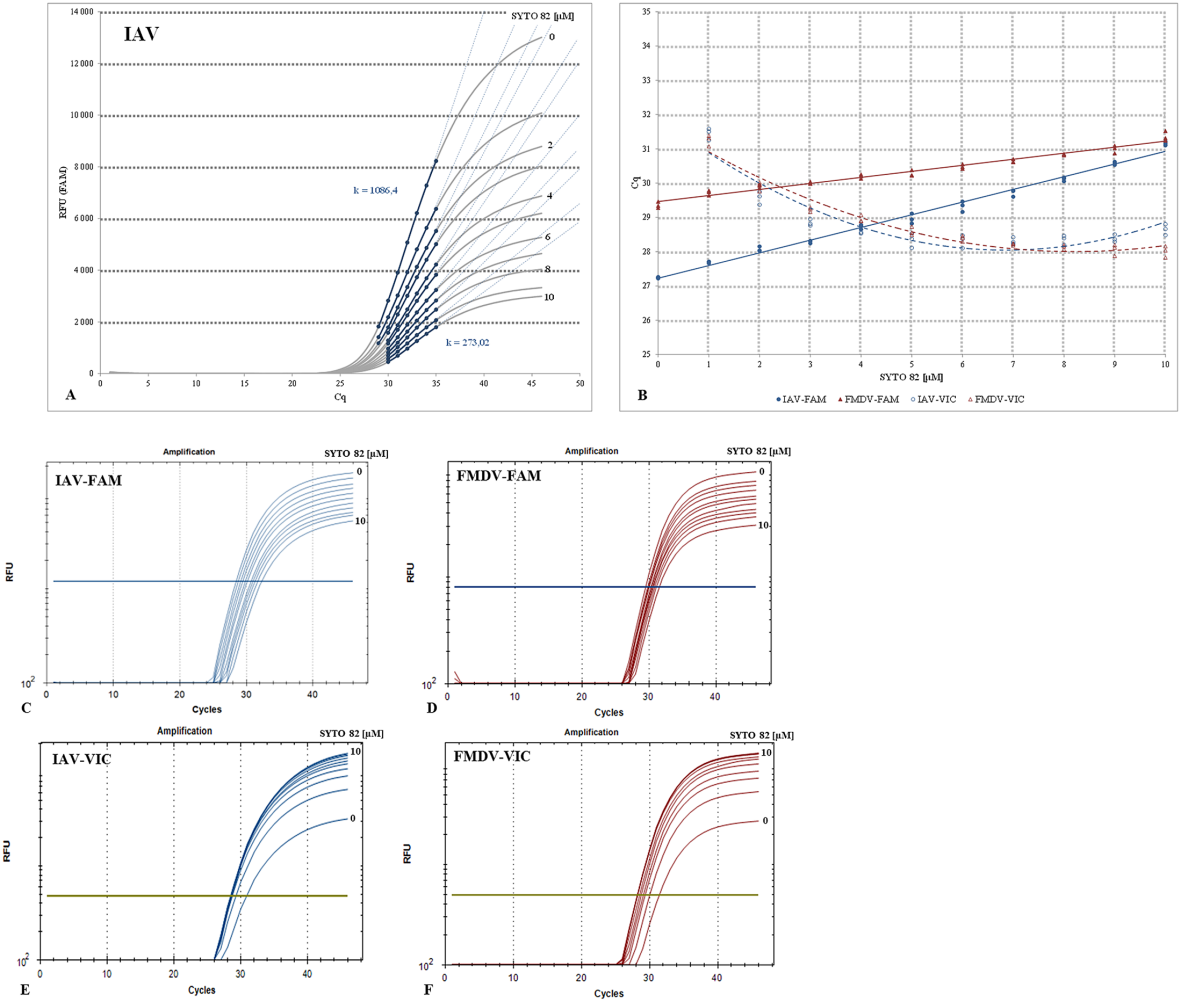
Effect of the SYTO 82 dye on the FAM-TaqMan assay. (A) The mean FAM d(RFU) data of the S82 gradient, ranges from 0 to 10μM, in 1μM increments, were shown for the IAV assay (Table 1) performed as a qPCR. Each amplification curve represents an average of three TaqMan reaction replicates per S82 concentration for a fixed initial synthetic DNA template amount of 1e4 copies/μl. The linear regression lines drawn across the exponential region of each amplification curve are dashed, and the region used for regression line construction is highlighted in blue. The slope values *k* were designated for the two S82 concentration extremities. For additional details, please refer to Table A and Fig BA in S2 File. (B), the Cq plot was constructed by plotting the Cq values of the IAV (blue) and FMDV (brown) assays against the S82 concentration. The data obtained from the FAM and VIC channels were designated with filled marks and solid lines or empty marks and dashed lines, respectively. (C-F) illustrate the FAM and VIC channel amplification curves of the IAV and FMDV assays in logarithmic views for one replicate series.

The evaluation of the S82 gradient showed that increasing concentration of the dye had a remarkable suppressive effect on the FAM fluorescence curve (Fig 1A; Table A and Fig BA in S2 File). To quantify the observed changes, the slope values, *k*, of the lines drawn along the linear region of the FAM curves were compared as a measure of the steepness of the curve. However, we used the linear phase of the sigmoidal curve instead of its logarithm [22]. The suppression was proportional to the dye concentration. The higher the S82 increment was in the reaction the more flat the amplification curves were which was reflected in decreasing *k* values. The FAM suppression was the most remarkable after comparing the two extreme dye concentration points (k = 1077.9 vs. 337.7 for the FMDV and k = 1086.4 vs. 273.02 fort the IAV assay). Nevertheless, the FAM curves retained the sigmoidal shape even at the highest S82 concentration tested.

To further characterise the S82 concentration dependent FAM signal changes, the d(RFU) parameter was employed enabling to compare the amplification curves at a specific point which corresponds to the first derivative maximum. A comparable strategy was used previously [23]; however, with the mathematical middle of the sigmoidal trajectory. Like in the previous point, plotting the FAM d(RFU) values in percentages against the S82 concentrations (Table A and Fig BB in S2 File) showed a continuous signal decline with a drop to only 25–30% of the original intensity at the extreme dye concentration point. The d(RFU) values between the 0 and 1μM differed by 21–23%_._

Similarly, the gradual increase in the concentration of S82 was accompanied by a slight but continuous increase in the FAM curve Cq values (Fig 1B). Although the changes in the Cq values were not as remarkable as those in the FAM fluorescence, the mean differences between 0 and 10μM oscillated around 2 for FMDV and 4cycles for IAV. Similar observations were made after Cq estimation directly from the fluorescence values by using the qPCR Miner [13], data not shown. Interestingly, the qPCR Miner did not reveal any consistent differences in reaction efficiencies throughout the S82 gradient (data not shown).

At first sight, the presented observations are in clear contradiction with the previously published results [10, 11]. To unravel this discrepancy, a re-evaluation of the amplification data was performed, and the FAM and VIC channel Cq values were compared again (Fig 1C–1F; Table A in S2 File). Since the melting analysis showed a single peak, the VIC curves were also indicative of a sequence-specific amplification. The FAM: VIC Cq comparison revealed inverse relationships. While the FAM curve Cq values decreased with an increased S82 concentration, the VIC Cq values were the highest at the lowest S82 concentration, then slightly decreased and from the concentration point of 3μM remained virtually identical (Fig 1B). Similarly, electrophoresis at the zero, five, and ten μM S82 concentration points of S82 showed comparable amplicon yields (Table B, and Figs C,B and CD in S2 File) which clearly suggested that the S82 dye was evidently not a physical inhibitor of amplification. This finding brings our data into line with the previous observations [10, 11] and was taken into account during the adjustment of the optimal S82 concentration.

So, which mechanism accounts for the FAM signal suppression? The projection of the FAM emission and S82 absorption spectra in a single graph revealed a voluminous overlap (Fig AB in S2 File; spectral overlap integral J = 3.693e+10 nm^4^M^-1^cm^-1^) in spite of the clearly distinct maxima. The FAM-S82 overlap is comparable to that of the FAM-TAMRA, a frequently used FAM quencher (J = 3.732e+10 nm^4^M^-1^cm^-1^). Indeed, the spectral overlap provides the most obvious explanation of the FAM curve suppression effect, i.e., via S82-dependent signal interference. In this case, we defined the interference as anything which modifies the FAM signal. Hence, the FAM-TaqMan reaction is apparently more S82 dye concentration sensitive than the qPCR using DNA binding dyes which tolerates higher dye amounts [10, 11]. However, other possible factors which could, theoretically, suppress the FAM signal, like inhibition of the TaqMan probe hydrolysis, could not have been ruled out.

### Influence of the SYTO 82 dye on the melting analysis

**Fig 2:**
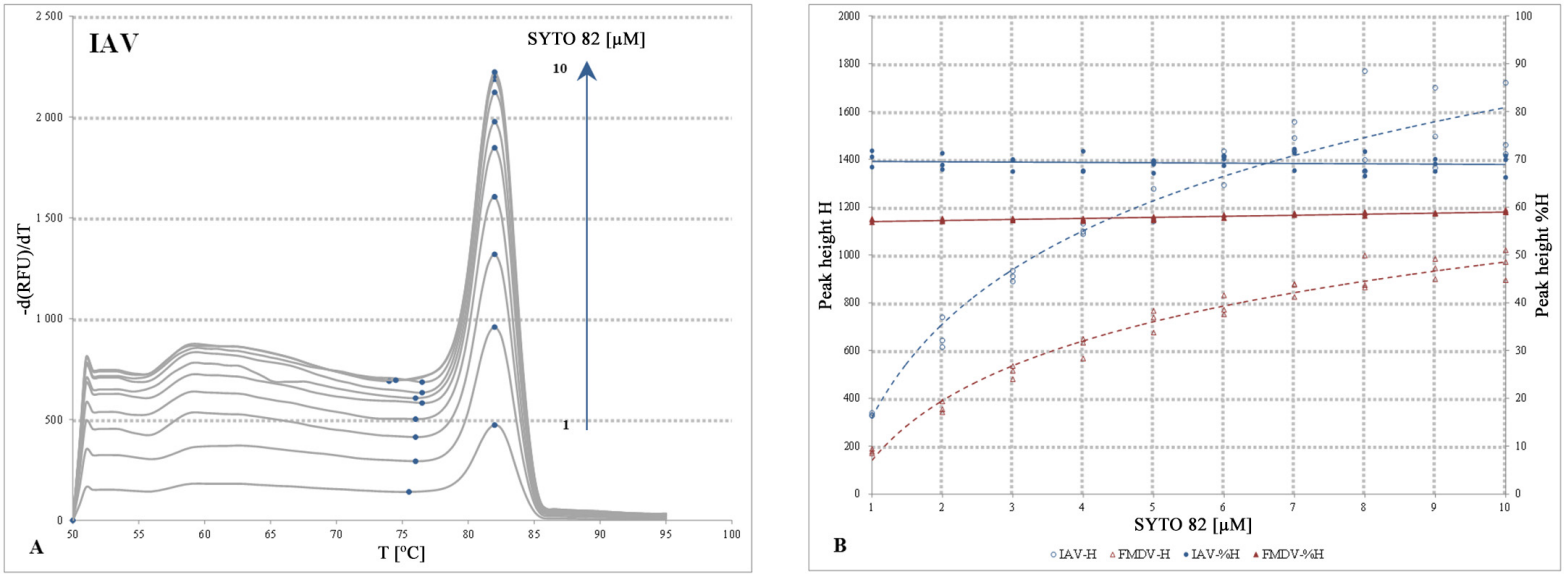
Effect of the SYTO 82 dye on the melting peak profiles. (A)The mean melting curve derivative data were shown for the IAV assay (Table 1) performed as a qPCR. Each melting peak represents an average of three replicates per S82 concentration with a fixed initial synthetic DNA template amount of 1e4 copies/μl. The S82 gradient, ranges from 1 to 10μM, in 1μM increments, was designated with a blue arrow. For each peak, the left tail local minimum and maximum values used for the estimation of the peak height H and proportionality, %H, were designated with blue spots. (B), the melting peak height and proportionality values plotted against the S82 concentration. The melting peak heights for the FMDV (brown, Fig D in S2 File) and IAV assays (blue) were designated with dashed lines and open symbols and related to the primary y axis. The secondary y axis indicates the %H values visualized by filled symbols and solid lines. For additional details, please refer to Table A in S2 File.

The same IAV and FMDV S82 gradients were subjected to melting analysis with signal acquisition in the VIC channel and the melting peak heights were evaluated. The peak heights represent a more interpretable parameter than the full width and half maximum (FWHM) or asymmetry values used for peak characterization elsewhere [24]. The melting analysis revealed only the amplicon-specific peaks, with a parallel alignment of peaks along the entire gradient (Fig 2A; Fig D in S2 File). No deviations in Tm were observed between the S82 reactions. Hence, S82 does not influence the Tm values at the tested concentrations in accordance with the previous observations [10, 11].

Higher S82 concentrations facilitated the melting peak evaluation by generating a stronger signal which was reflected in the absolute melting peak heights H where the two extremities differed by ~80% (Fig 2A and 2B; Table A in S2 File). As was further shown in Fig 2B, the positive correlation between the concentration of S82 and H values can be seen for the range of 1–6μM with a plateau recognisable from the point of 8μM.

Despite the marked differences between the peak heights, the peak proportionality values, estimated as the background-to-tip ratio in percentages (Fig 2B), were highly similar across the gradient and differed only by 1.2% for the IAV and 1.9% for the FMDV assays. This proved that the peak proportionality was retained along the entire gradient and even the lowest S82 dye amounts could provide clearly interpretable full-fledged melting profiles.

### Determination of the optimal SYTO 82 dye concentration in the TaqMan assay

As was shown, the determination of the optimal S82 concentration in the TaqMan qPCR is generally influenced by two opposing factors: i) the optimal concentration should be sufficiently low to minimize the spectral interference, but ii) it should also be high enough to ensure sensitive melting peak analysis. Previous reports have suggested that the qPCR utilizing DNA binding dyes can tolerate two or as much as 10μM of S82 without a noticeable influence on the reaction parameters [10, 11]. However, our data indicate that the TaqMan qPCR is more S82 sensitive which could be attributed mainly to the signal interference. Hence, even the lowest amount tested of S82 meant 21–23% FAM suppression. Such drop in the specific signal might impede the interpretation of weak positive specimens which implies that the optimal S82 concentration should be further decreased to less than 1μM. On the other hand, using submicromolar amounts of S82 led to decreased sensitivity of the melting analysis, with fuzzy peaks observed especially for weaker specimens (data not shown). This effect is, however, greatly influenced by the optics of the qPCR platform used (data not shown).

Low dye concentrations can theoretically promote additional undesirable effects arising from the variation in amplicon length or population size and from the competition between the specific and nonspecific reaction products differing in the length and abundance. S82 is considered rather as a lower affinity DNA binding dye [21] which would theoretically tend to exacerbate the detection of short amplicons as had been observed in Eva Green [25]. The shorter the amplicon, the smaller the number of dye interacting sites. This may, in the case of a small population of short amplicons, result in a less bright signal [25, 26] impairing the sensitivity of the melting analysis. Finally, the generation of undesired products may, in the case of limiting dye amounts, drain out a significant dye fraction due to the competition with the specific amplicons or relocation [27–29] leading to inconsistent melting patterns. This effect is more important when the unspecific products are longer and/or more abundant than the specific amplicons.

So, is it possible to reach the optimal S82 amount in the TaqMan assay? Could we minimize or compensate for the FAM suppressive effect while retaining sufficient sensitivity of the melting analysis? Which dye concentration should be used to achieve the best possible results? Can we use the same amount of S82 universally across different qPCR assays?

The above assumptions imply that the optimization of the MeltMan qPCR would require mutual adjustment of the S82 and hydrolysis probe ratio rather than lowering the dye concentration itself. A similar approach was considered previously for the optimization of the FAM and VIC probe ratio [30]. To this end, various FAM probe: S82 ratios were tested in different qPCR assays (data not shown). The results suggested that there exists an optimal dye: probe ratio that, under the conditions used, sufficiently compensates for the spectral interference and ensures acceptable sensitivity of the melting analysis. This ratio was established to be 0.8μM S82: 0.4μM FAM probe and was further evaluated.

### Evaluation of the reaction parameters of the MeltMan system

According to the MIQUE guidelines [2], the evaluation of the MeltMan system containing 0.4μM of the probe and 0.8μM of S82 was based on the calibration curve analysis of the FMDV and IAV assays. To provide more detailed characterization, the data were supplemented with the estimated *k*, d(RFU), and H values.

**Fig 3:**
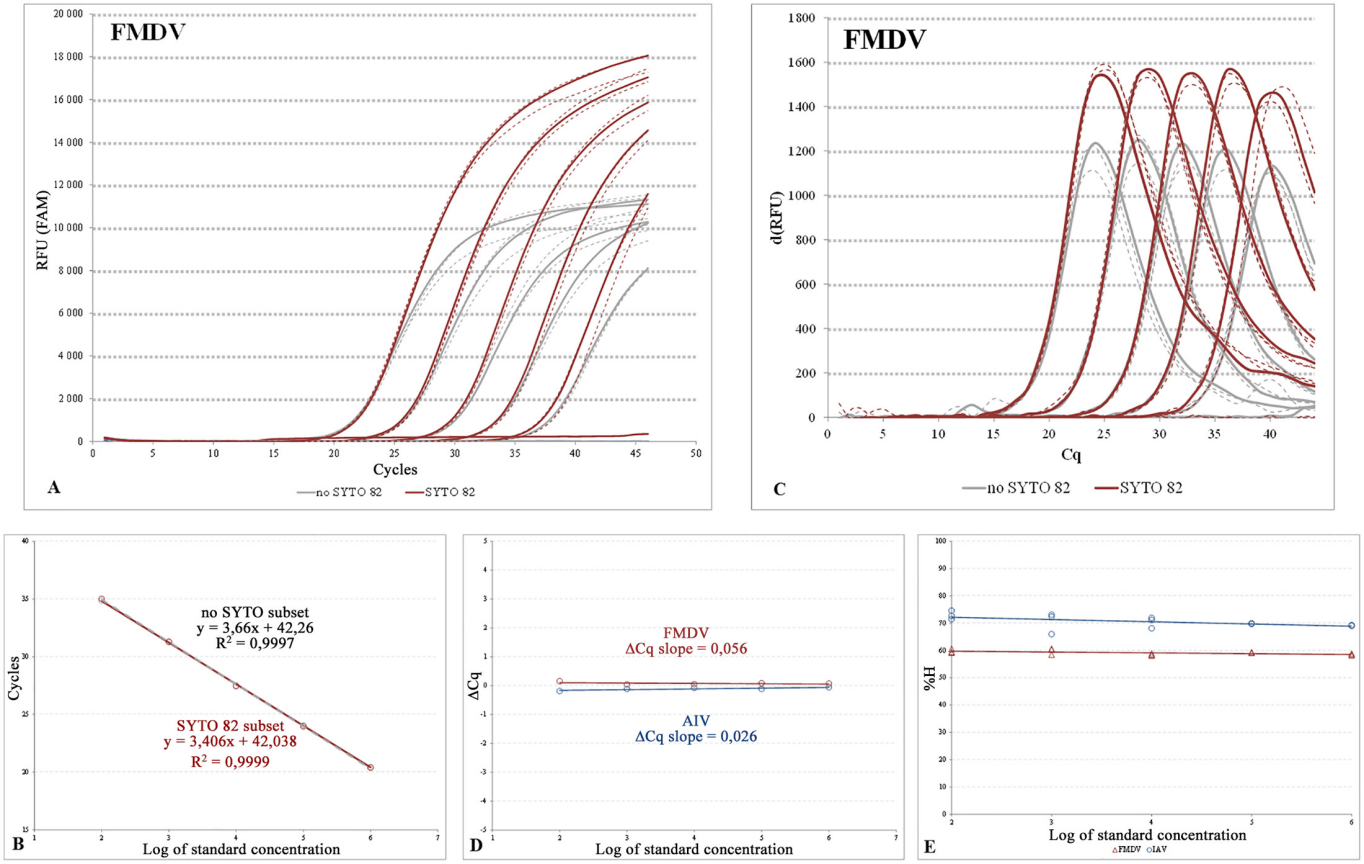
Evaluation of the MeltMan assay. The concentration gradient of the FMDV qPCR (Table 1) from 1e2 to 1e6 synthetic DNA template copies/μl in three replicates (A) and the calibration curves (B). For the amplification and calibration curves of the IAV assay please refer to Figs EA and EB in S2 File. The results for no S82 and S82 subsets were highlighted in grey and brown, respectively. For clarity, certain curves were visualized as dashed. (C) the d(RFU) FAM data. (D and E) the ΔCq and %H plots for the FMDV (brown), and IAV (blue) assays, respectively. The data are corresponding with Table C in S2 File.

As seen in Fig 3A (Fig EA in S2 File), the early exponential phases of the corresponding amplification curves for the subsets tested were tightly overlapped across the entire dilution range. This resulted in highly similar Cq values in both assays and was also reflected in the perfectly parallel and overlapping standard curves with almost identical efficiency, slope, and R^2^ values (Fig 3B; Fig EB and Table C in S2 File).

In relative quantification, the standard and test subsets resembled the target and reference gene assays. Therefore, as an additional evaluation approach, it was possible to implement the ΔCq line slope strategy [31] to compare the reaction efficiencies. When the ΔCq values of the corresponding standard and S82 subsets were plotted against the log of concentration, the ΔCq plot revealed slope values of 0.056 for the FMDV and 0.026 for the IAV assays which is markedly below the 0.1 threshold (Fig 3C). This proved that the reaction efficiencies of the subsets are comparable.

However, as the amplification proceeded, the initially overlapping curves started to when entering the linear phase which remarkably changed the resulting amplification profile where the S82 subsets showed a steeper slope in comparison to the standard assays (Table C in S2 File). Similarly, the d(RFU) values in percentages (Fig 3D; Fig EC and Table C in S2 File) revealed that the S82 subsets had consistently higher FAM signal intensity.

Finally, the evaluation of the melting peaks of the S82 subsets in the VIC channel showed that the 0.8μM concentration of S82 allows clear melting peak visualization and interpretation, even at the lowest standard concentration point. When comparing the H values in percentages, the melting peaks exhibited relatively equal height throughout the entire dilution range (Fig 3E; Figs ED, EE and Table C in S2 File).

The results proved that the 0.8μM dye: 0.4μM probe ratio sufficiently compensated for the FAM signal loss and improved the emitted FAM signal intensity, yielding consistently steeper amplification curves while keeping the remaining reaction parameters unaffected. In addition, the 0.8μM amount of S82 allowed sufficiently sensitive and reproducible melting analysis with virtually equal melting peak heights across the entire dilution range used.

### Evaluation of the sensitivity of the MeltMan system

As shown above, the 0.4μM probe: 0.8μM S82 ratio was compatible with the TaqMan qPCR. Therefore, in the next step, we investigated whether and how S82 influences the sensitivity of amplification and melting analysis under real conditions using field specimens. To this end, the parametric differences (Cq, d(RFU) FAM, and H) between the standard and test subsets were analysed in seven different TaqMan assays. To inspect the possible amplicon length effects, the assays were selected to represent short (<100bp EHV-1 [14], celery DNA [16], chicken DNA [17], and TBEV [18]), medium (>100 and <200bp, IAV [7] and OvHV-2 [15]), and long (>200bp EAV [19]) amplicons (Table 1). To investigate the system behaviour at the extreme end of the amplification (Cq >30 cycles), we used diluted and unquantified field specimens and evaluated them in ten replicates per subset.

**Fig 4:**
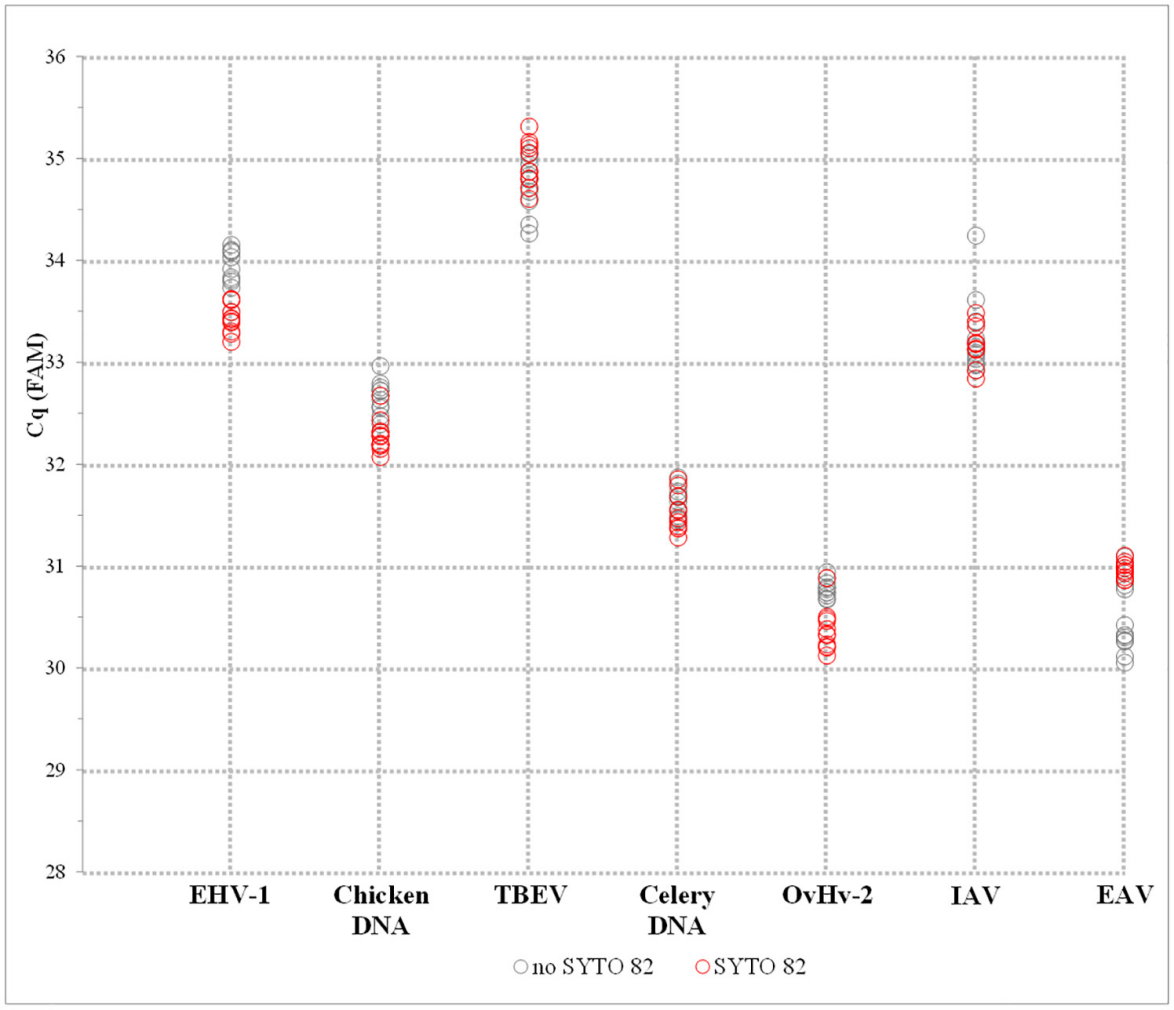
Sensitivity of the MeltMan reaction system. Seven TaqMan assays: Chicken DNA, EHV-1, TBEV, OvHV-2, IAV, EAV, and Celery DNA (Table 1) were analysed by using diluted field specimens to reach Cq values of ≥30. The assays were prepared in two subsets, no S82 (grey) and S82 (red), with ten replicates per subset. The graph represents the Cq values obtained for each particular replicate. The data are corresponding with Fig F and Table D in S2 File.

The results of all seven TaqMan assays are summarised in Table D and Fig F in S2 File. Overall, no significant differences were observed between the standard and test subsets, with both yielding highly similar Cq values (Fig 4) and low standard deviation values suggesting low intra-assay or intra-subset variation.

The evaluation of the FAM d(RFU) data showed improved signal for the majority of S82 subsets (Fig F and Table D in S2 File). Although, for certain assays, higher d(RFU) dispersions were observed, the data consistently showed no difference or improved FAM signal strength.

Finally, the analysis of the melting curves in the VIC channel revealed clearly interpretable and reproducible melting peaks, supported by comparable peak heights with low standard deviation values observed within all ten reaction replicates independently of amplicon length and NA template type (Fig F and Table D in S2 File).

The evaluation of the MeltMan system using weak field specimens clearly proved that the tested FAM probe: S82 ratio does not influence the sensitivity of the TaqMan qPCR or RT-qPCR assay and has no observable inhibitory effect on amplification or reverse transcription. Similarly, the melting peak data suggested that the selected S82 concentration is sufficient for a clear and reproducible melting analysis and peak interpretation of weak positive RNA or DNA templates of various amplicon lengths.

### Compatibility of the SYTO 82 dye with various TaqMan assays

A total of 18 qPCR and 18 RT-qPCR assays were evaluated using simple, MGB and LNA modified probes (Table 1). The templates included a variety of specimens as well as pure virus or bacterial cultures (8≤N≤55). For each specimen, the S82 and no S82 tests were run in parallel and the Cq and FAM d(RFU) values were statistically evaluated separately for DNA and RNA assays (Table 1). In addition, all of the assays were subjected to melting analysis and the melting profiles of interest were further refined by electrophoresis. The statistical evaluation was based on the data obtained by the CFX96 instrument using the QuantiTect and AgPath-ID OneStep RT-PCR (Life Technologies) kits. In addition, the MiniOpticon (BioRad), LC480 (Roche), and Rotorgene Q (Qiagen) platforms were also evaluated (not shown).

**Fig 5:**
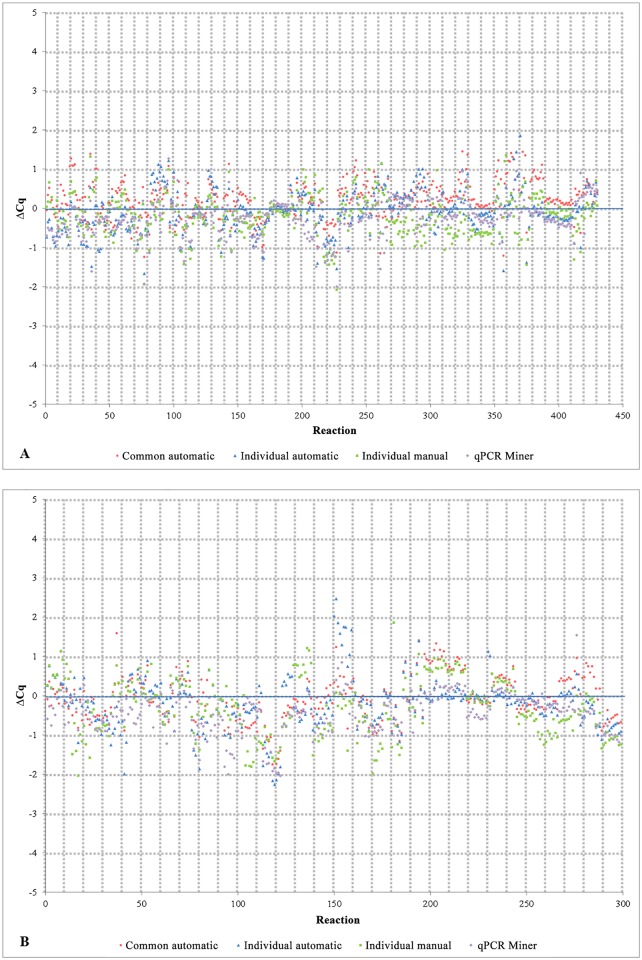
Compatibility of the S82 dye with various TaqMan assays. The qPCR (A) and RT-qPCR ΔCq scatter plots (B) show the distribution of the ΔCq values per each S82 and no S82 paired reaction obtained by four different baseline approaches. The frequencies of the particular ΔCq values are provided in Figs GC and GD in S2 File.

The qPCR ΔCq scatter plot, summarizing a total of 430 paired assays, showed relatively equal distribution of the data with the majority of points spanned between ±1 cycle (Fig 5A). Despite the fact that the four baseline approaches gave different Cq values, the no S82 and S82 subsets were highly similar (p<0.001). This was also reflected in the slope of the straight lines plotted across the particular ΔCq values (*k* ≤0.011) which again suggested high correlation. Similarly, albeit sparser, the RT-qPCR ΔCq scatter plot, counting 299 data pairs, (Fig 5B) revealed the distribution of the data within the interval of ±2 cycles and ΔCq plot slopes *k* of ≤0.013. These data proved that S82 in a 0.8μM concentration is compatible with all of the tested assays and does not influence the Cq values in a statistically significant manner. However, a more detailed investigation of the results may suggest differences in the tolerance of the 0.8μM concentration of S82 between assays. For example, the IAV S82 subset yielded slightly higher Cq values than the no S82 subset. On the contrary, other assays like the AIV [32] or the AIV-H9 [33] showed no differences at all. Although the observed differences were not statistically significant and could be probably attributed to pipetting errors, the suboptimal performance of the 0.8μM S82 concentration in some assays cannot be fully ruled out due to the small number of assays evaluated.

In the next step the d(RFU) and %d(RFU) values of the FAM curve were compared between the no S82 and S82 subsets. The %d(RFU) plot (Figs GA and GB in S2 File) showed a predominant distribution of the data above zero for both datasets. Similarly, the comparison of the mean d(RFU) values did not find statistically significant differences between the subsets (p<0.001). This demonstrates that the 0.4μM FAM probe concentration sufficiently compensated for the S82 dependent signal loss in the majority of assays.

Finally, evaluation of the melting peaks in the VIC channel provided a plethora of various melting profiles where the most interesting ones, especially those with multiple nonspecific amplicons, were correlated with electrophoretic patterns. Several assays generated nonspecific amplicons or primer dimers. This partly resulted from the reaction conditions used, e.g., the fixed primer concentration, high number of cycles, etc., which might have been suboptimal for certain assays. Nevertheless, such reaction profiles could be used to investigate the sensitivity, discernibility, or dye relocation effects [25, 28, 29]. Three representative melting profiles were selected for illustration. The sensitivity and dye relocation were tested by ten repeated melting analyses of the reactions showing two different amplicon population sizes (Profile 5A in S1 File) and the discernibility was investigated on reactions generating multiple amplicons (Profile 5B in S1 File). As a result the melting analysis using 0.8μM S82 revealed sufficient sensitivity of the system which apparently goes beyond the results of chapter 3.6. In addition, no dye relocation was observed. On the other hand, the melting peak discernibility is greatly influenced by amplicon Tm values. The closer the Tm values the less discernible the neighbouring peaks. These peaks could be merged into a single wide and flat peak and the products can be discriminated only by electrophoresis.

Taken together, the presented results clearly proved that the tested S82 and FAM probe concentration did not significantly influenced the qPCR or RT-qPCR assays using “classical“, MGB or LNA modified FAM probes and are sufficient to detect amplicons of various lengths and abundance even in the same assay. On the other hand, slight differences in the S82 toleration between certain assays and in the sensitivity between qPCR platforms were indicated. Therefore, the 0.8μM S82:0.4μM probe should rather serve as the upper starting point for the selection of the most appropriate concentration for a given assay or qPCR instrument. Similarly, the S82concentration should be adjusted in dependence on the analysis concept. When the aim is only the specific amplicon confirmation, the S82 concentration can be further decreased along with setting the starting melting temperature to a few degrees below the specific Tm to ensure as high free S82 molecules as possible. On the other hand, for the investigation of the entire amplicon population the S82 concentrations around 0.8μM should be used. Hence, the full implementation of the MeltMan system in routine use would require more comprehensive validation under a given laboratory settings including a broader panel of specimens along with various threshold estimation approaches.

The idea to integrate the TaqMan and melting analysis into a single assay is not entirely new [34–36]. In 2006, Lind and colleagues [34] were the first to present a system combining the FAM-labeled TaqMan probes with the BOXTO dye that allowed parallel signal processing in the green and yellow channels, respectively. The two remaining works relied on SG, ROX, and Cy5 [35] or FAM, HEX, TexasRed, and Cy5 [36] probe labels. Although proven fully functional, these techniques were designed for specific applications [35, 36], and their potential for broader use has not yet been evaluated [34]. Based on the studies comparing the performance of multiple DNA dyes in qPCR [10, 11], we took advantages of the S82 DNA binding dye to integrate the FAM-TaqMan and melting analysis into a single assay. This approach utilises the two most popular detection channels, green and yellow, which allows the use of the existing FAM probes of our TaqMan assay library without the necessity of re-ordering probes with different fluorescent labels. The presented technique was evaluated in 36 different qPCR assays including 729 paired reactions performed in four qPCR laboratories.

In diagnostic microbiology capturing the melting analysis with TaqMan qPCR allow the evaluation of specific amplicons along with the corresponding melting peaks in a single assay, which enhances the confidence of the analysis. The MeltMan system would also broaden the inclusivity of the assay by precluding molecular diagnostic drawbacks resulting from reduced or abolished probe binding due to mutations in the corresponding template regions [5–9]; Profiles 1A-D in S1 File). Further, it may help to solve the problems arising from weird FAM curves (Profiles 2and 3 in S1 File) without the need to repeat the analysis. The evaluation of the melting profiles could be helpful in detecting cross amplification (Profiles 4A and 4B in S1 File) or investigating the entire assay background (Profile 5B in S1 File) and has potential for the detection of new taxons, genetic variants, phylogenetic lineages or discrimination between the pathotypes. Last but not least, the S82-TaqMan system can be a useful tool in the development and evaluation of new TaqMan assays and can open up the scope for advanced melting peak analyses [37, 38] or, theoretically, for high resolution melting [39] in the MeltMan qPCR.

Despite the undisputable advantages of the MeltMan approach in diagnostic qPCR, the system displays also several drawbacks which could limit its application. First of all, multiple melting peaks generated during the analysis may be confusing rather than conclusive. In an extreme case, a nonspecific product in negative reactions may have a melting peak with a very close or even identical Tm to that of the positive control which can be interpreted as false positivity. Further, the already mentioned discriminatory power of the melting analysis could be an issue especially when the specific and nonspecific amplicons are generated alongside in the same reaction. Both of these phenomena were observed during the evaluation (data not shown). However, the generation of such byproducts is an inherent property of the assay used, which could be, to some extent, influenced by the settings used in a given laboratory. Additional disadvantages are higher probe consumption or extended analysis time.

Nevertheless, linking the TaqMan and melting analysis into a single optimized reaction system provides the opportunity to perform more comprehensive analyses, get more data from the investigated specimens, and obtain qPCR results with higher confidence.

## Supporting Information

S1 File MeltMan application examples. (PDF)

S2 File Contains supporting Figs A-G and Tables A-D. (PDF)

## References

[1] Saiki RK, Scharf S, Faloona F, Mullis KB, Horn GT, Erlich HA, et al Enzymatic amplification of beta-globin genomic sequences and restriction site analysis for diagnosis of sickle cell anemia. Science. 1985; 230: 1350–54. 2999980 10.1126/science.2999980

[2] Bustin SA, Benes V, Garson JA, Hellemans J, Huggett J, Kubista M, et al The MIQE Guidelines: Minimum Information for Publication of Quantitative Real-Time PCR Experiments. Clin Chem. 2005; 55: 611–622. 10.1373/clinchem.2008.112797 19246619

[3] Mackay IM, Mackay JF, Nissen MD, Sloots TP. Real-time PCR: History and fluorogenic chemistries In: Mackay IM, editor. Real-time PCR in microbiology. Norfolk: Caister Academic Press; 2007, pp. 1–39.

[4] Holland PM, Abramson RD, Watson R, Gelfand DH. Detection of specific polymerase chain reaction product by utilizing the 5'——3' exonuclease activity of *Thermus aquaticus* DNA polymerase. Proc Natl Acad Sci USA. 1991; 88: 7276–7280. 1871133 10.1073/pnas.88.16.7276 PMC52277

[5] Papin JF, Vahrson W, Dittmer DP. SYBR green-based real-time quantitative PCR assay for detection of West Nile Virus circumvents false-negative results due to strain variability. J Clin Microbiol. 2004; 42: 1511–1518. 15070997 10.1128/JCM.42.4.1511-1518.2004 PMC387603

[6] Cattoli G, De Battisti C, Marciano S, Ormelli S, Monne I, Terregino C, et al False-negative results of a validated real-time PCR protocol for diagnosis of newcastle disease due to genetic variability of the matrix gene. J Clin Microbiol 2009; 47: 3791–3792. 10.1128/JCM.00895-09 19759219 PMC2772626

[7] Nagy A, Vostinakova V, Pirchanova Z, Černíková L, Dirbáková Z, Mojžiš M, et al Development and evaluation of one step real-time RT-PCR assay for universal detection of influenza A viruses from avian and mammal species. Arch Virol. 2010; 155: 665–673. 10.1007/s00705-010-0636-x 20229116 PMC7086820

[8] Garson JA, Ferns RB, Grant PR, Ijaz S, Nastouli E, Szypulska R, et al Minor groove binder modification of widely used TaqMan probe for hepatitis E virus reduces risk of false negative real-time PCR results. J Virol Methods. 2012; 186: 157–160. 10.1016/j.jviromet.2012.07.027 22871672

[9] Armstrong PM, Prince N, Andreadis TG. Development of a multi-target TaqMan assay to detect eastern equine encephalitis virus variants in mosquitoes. Vector Borne Zoonotic Dis. 2012; 12: 872–876. 10.1089/vbz.2012.1008 22835151 PMC3466067

[10] Gudnason H, Dufva M, Bang DD, Wolff A. Comparison of multiple DNA dyes for real-time PCR: effects of dye concentration and sequence composition on DNA amplification and melting temperature. Nucleic Acids Res. 2007; 35: e127 17897966 10.1093/nar/gkm671 PMC2095797

[11] Eischeid AC. SYTO dyes and EvaGreen outperform SYBR Green in real-time PCR. BMC Research Notes. 2011; 4: 263 10.1186/1756-0500-4-263 21798028 PMC3162529

[12] Reid SM, Ferris NP, Hutchings GH, Zhang Z, Belsham GJ, Alexandersen S. Detection of all seven serotypes of foot-and-mouth disease virus by real-time, fluorogenic reverse transcription polymerase chain reaction. J Virol Methods. 2002; 105: 67–80. 12176143 10.1016/s0166-0934(02)00081-2

[13] Zhao S, Fernald RD. Comprehensive algorithm for quantitative real-time polymerase chain reaction. J Comput Biol. 2005; 12: 1045–62. Available: http://ewindup.info/miner/index.htm 10.1089/cmb.2005.12.1047 PMC2716216 16241897

[14] Diallo IS, Hewitson G, Wright LL, Kelly MA, Rodwell BJ, Corney BG. Multiplex real-time PCR for the detection and differentiation of equid herpesvirus 1 (EHV-1) and equid herpesvirus 4 (EHV-4). Vet Microbiol 2007; 123: 93–103. 17346907 10.1016/j.vetmic.2007.02.004

[15] Traul DL, Taus NS, Oaks JL, O’Toole D, Rurangirwa FR, Baszler TV, et al Validation of nonnested nad real-time PCR for diagnosis of sheep-associated malignant catarrhal fever in clinical samples. J Vet Diagn Invest. 2007; 19: 405–408. 17609352 10.1177/104063870701900412

[16] Ch Hupfer, Waiblinger H-U, Busch U. Development and validation of a real-time PCR detection method for celery in food. Eur Food Res Technol. 2007; 225: 329–335.

[17] Köppel R, Zimmerli F, Breitenmoser A. Heptaplex real-time PCR for the identification and quantification of DNA from beef, pork, chicken, turkey, horse, meat, sheep (mutton) and goat. Eur Food Res Technol. 2009; 230: 125–133.

[18] Achazi K, Nitsche A, Patel P, Radoni A, Donoso Mantke O, et al Detection and differentiation of tick-borne encephalitis virus subtypes by a reverse transcription quantitative real-time PCR and pyrosequencing. J Virol Methods. 2011; 171: 34–39. 10.1016/j.jviromet.2010.09.026 20933016

[19] Balasuriya UBR, Leutenegger ChM, Topol JB, McCollum WH, Timoney PJ, MacLachlan NJ. Detection of equine arteritis virus by real-time TaqMan^®^ reverse transcription-PCR assay. J Virol Methods. 2002; 101: 21–28. 11849680 10.1016/s0166-0934(01)00416-5

[20] Molecular Probes. SYTO orange fluorescent nucleic acid strains. Product information brochure. 2001 Available: https://tools.lifetechnologies.com/content/sfs/manuals/mp11360.pdf

[21] The Molecular Probes Handbook. Available: https://www.lifetechnologies.com/cz/en/home/references/molecular-probes-the-handbook/nucleic-acid-detection-and-genomics-technology/nucleic-acid-stains.html#head6

[22] Ruijter JM, Ramakers C, Hoogaars WMH, Karlen Y, Bakker O, van den Hoff MJB, et al Amplification efficiency: linking baseline and bias in the analysis of quantitative PCR data. Nucleic Acids Res. 2009; 37: e45 10.1093/nar/gkp045 19237396 PMC2665230

[23] Rutledge RG, Stewart D. A kinetic-based sigmoidal model for the polymerase chain reaction and its application to high-capacity absolute quantitative real-time PCR. BMC Biotechnol. 2008; 8: 47 10.1186/1472-6750-8-47 18466619 PMC2397388

[24] Hinshaw J. Anatomy of a peak. GC North America. 2004; 3: 252–260.

[25] Mao F, Leung WY, Xin X. Characterization of EvaGreen and the implication of its physicochemical properties for qPCR applications. BMC Biotechnology. 2007; 7: 76 17996102 10.1186/1472-6750-7-76 PMC2213645

[26] Colborn JM, Byrd BD, Koita OA, Krogstad DJ. Estimation of copy number using SYBR Green: confounding by AT-rich DNA and by variation in amplicon length. Am J Trop Med Hyg. 2008; 79: 887–892. 19052298

[27] Giglio S, Monis PT, Saint CP. Demonstration of preferential binding of SYBR Green I to specific DNA fragments in real-time multiplex PCR. Nucleic Acids Res. 2003; 15: e136. 10.1093/nar/gng135 PMC275573 14602929

[28] Monis PT, Giglio S, Saint CP. Comparison of SYTO9 and SYBR Green I for real-time polymerase chain reaction and investigation of the effect of dye concentration on amplification and DNA melting curve analysis. Anal Biochem. 2005; 340: 24–34. 15802126 10.1016/j.ab.2005.01.046

[29] Varga A, James D. Real-time RT-PCR and SYBR Green I melting curve analysis for the identification of Plum pox virus strains C, EA, and W: effect of amplicon size, melt rate, and dye translocation. J Virol Methods. 2006; 132: 146–53. 16293321 10.1016/j.jviromet.2005.10.004

[30] Lee DH, Mathew J, Pfahler W, Ma D, Valinsky J, Prince AM, et al Individual donor nucleic acid amplification testing for detection of West Nile virus. J Clin Microbiol. 2005; 43: 5111–5116. 16207971 10.1128/JCM.43.10.5111-5116.2005 PMC1248480

[31] Qiagen. Critical factors for successful real-time PCR. Real-Time PCR Brochure. 2010; 07: 1–63. Available: https://www.qiagen.com/cz/resources/resourcedetail?id=f7efb4f4-fbcf-4b25-9315-c4702414e8d6&lang=en

[32] Spackman E, Senne DA, Myers TJ, Bulaga LL, Garber LP, Perdue ML, et al Development of a real-time reverse transcriptase PCR assay for type A influenza virus and the avian H5 and H7 hemagglutinin subtypes. J Clin Microbiol. 2002; 40: 3256–3260. 12202562 10.1128/JCM.40.9.3256-3260.2002 PMC130722

[33] Monne I, Ormelli S, Salviato A, De Battisti C, Bettini F, Salomoni A, et al Development and validation of a one-step real-time PCR assay for simultaneous detection of subtype H5, H7, and H9 avian influenza viruses. J Clin Microbiol. 2008; 46: 1769–1773. 10.1128/JCM.02204-07 18367569 PMC2395090

[34] Lind K, Ståhlberg A, Zoric N, Kubista M. Combining sequence-specific probes and DNA binding dyes in real-time PCR for specific nucleic acid quantification and melting curve analysis. Biotechniques. 2006; 40: 315–319. 16568820 10.2144/000112101

[35] Cheah ES, Malkin J, Free RC, Lee SM, Perera N, Woltmann G, et al A two-tube combined TaqMan/SYBR Green assay to identify mycobacteria and detect single global lineage-defining polymorphisms in Mycobacterium tuberculosis. J Mol Diagn. 2010; 12: 250–256. 10.2353/jmoldx.2010.090030 20093392 PMC2871733

[36] Van Poucke M, Van Zeveren A, Peelman LJ. Combined FAM-labeled TaqMan probe detection and SYBR green I melting curve analysis in multiprobe qPCR genotyping assays. Biotechniques. 2012; 52: 81–86. 10.2144/000113808 22313405

[37] Mergny JL, Lacroix L. Analysis of thermal melting curves. Oligonucleotides. 2003; 13: 515–537. 15025917 10.1089/154545703322860825

[38] Mehndiratta M, Palanichamy JK, Ramalingam P, Pal A, Das P, Sinha S, et al Fluorescence acquisition during hybridization phase in quantitative real-time PCR improves specificity and signal-to-noise ratio. Biotechniques. 2008; 45: 625–626. 19238793 10.2144/000112994

[39] Liu Y, Tang J, Wakamatsu P, Xue H, Chen J, Gaynon PS, et al High-resolution melting curve analysis, a rapid and affordable method for mutation analysis in childhood acute myeloid leukemia. Front Pediatr. 2014; 2: 96 10.3389/fped.2014.00096 25250304 PMC4158872

[40] Decaro N, Desario C, Lucente MS, Amorisco F, Campolo M, Elia G, et al Specific identification of feline panleukopenia virus and its rapid differentiation from canine parvoviruses using minor groove binder probes. J Virol Methods. 2008; 147: 67–71. 17850892 10.1016/j.jviromet.2007.08.006

[41] Köppel R, Ruf J, Rentsch J. Multiplex real-time PCR for the detection nad quantification of DNA from beef, pork, horse and sheep. Eur Food Res Technol. 2011; 232: 151–155.

[42] European Union Reference Laboratory for Animal Proteins in Feedingstuffs. Detection of horse DNA using real-time PCR. EURL-AP. 2013; Version 1.0, 18.02.2013. Available: http://www.innofoodsee.eu/downloads/protocol_detection_horse_dna_using.pdf

[43] Evans JJ, Wictum EJ, Penedo CT, Kanthaswamy S. Real-time polymerase chain reaction quantification of canine DNA. J Forensic Sci. 2007; 52: 93–96. 17209917 10.1111/j.1556-4029.2006.00305.x

[44] King DP, Reid SM, Hutchings GH, Grierson SS, Wilkinson PJ, Dixon LK, et al Development of a TaqMan^®^ PCR assay with internal amplification control for the detection of African swine fever virus. J Virol Methods. 2003; 107: 53–61. 12445938 10.1016/s0166-0934(02)00189-1

[45] Fernández-Pinero J, Gallardo C, Elizalde M, Robles A, Gómez C, Bishop R, et al Molecular diagnosis of African swine fever (ASF) by a new real-time PCR using Universal Probe Library (UPL). Transbound Emerg Dis. 2013; 60: 48–58. 10.1111/j.1865-1682.2012.01317.x 22394449

[46] Tomaso H, Scholz HC, Al Dahouk S, Eickhoff M, Treu TM, Wernery R, et al Development of a 5'-nuclease real-time PCR assay targeting fliP for the rapid identification of *Burkholderia mallei* in clinical samples. Clin Chem. 2006; 52: 307–310. 16449212 10.1373/clinchem.2005.059196

[47] Thibault FM, Valade E, Vidal DR. Identification and discrimination of Burkholderia pseudomallei, B. mallei, and B. thailandensis by real-time PCR targeting type III secretion system genes. J Clin Microbiol. 2004; 42: 5871–5874. 15583328 10.1128/JCM.42.12.5871-5874.2004 PMC535269

[48] Abril C, Engels M, Liman A, Hilbe M, Albini S, Franchini M, et al Both viral and host factors contribute to neurovirulence of bovine herpesviruses 1 and 5 in interferon receptor-deficient mice. J Virol. 2004; 78: 3644–3653. 15016885 10.1128/JVI.78.7.3644-3653.2004 PMC371052

[49] Nitsche A, Buttner M, Wilhelm S, Pauli G, Mayer H. Real-time PCR detection of Parapoxvirus DNA. Clin Chem. 2006; 52: 316–319. 16449215 10.1373/clinchem.2005.060335

[50] Gilad O, Yun S, Zagmutt-Vergara FJ, Leutenegger ChM, Bercovier H, Hedrick RP Concentrations of a Koi herpesvirus (KHV) in tissues of experimentally infected *Cyprinus caprio koi* as assessed by real-time Taqman PCR. Dis Aquat Org. 2004; 60: 179–187. 15521316 10.3354/dao060179

[51] Everett KD, Hornung LJ, Andersen AA. Rapid detection of the Chlamydiaceae and other families in the order Chlamydiales: three PCR tests. J Clin Microbiol. 1999; 37: 575–580. 9986815 10.1128/jcm.37.3.575-580.1999 PMC84475

[52] Störmer M, Vollmer T, Henrich B, Kleesiek K, Dreier J. Broad-range real-time PCR assay for the rapid identification of cell-line contaminants and clinically important mollicute species. Int J Med Microbiol. 2009; 299: 291–300. 10.1016/j.ijmm.2008.08.002 18926769

[53] Letellier C, Kerkhofs P. Real-time PCR for simultaneous detection and genotyping of bovine viral diarrhea virus. J Virol Methods. 2003; 114: 21–27. 14599675 10.1016/j.jviromet.2003.08.004

[54] Callahan JD, Brown F, Osorio FA, Sur JH, Kramer E, Long GW, et al Use of a portable real-time reverse transcriptase-polymerase chain reaction assay for rapid detection of foot-and-mouth disease virus. J Am Vet Med Assoc. 2002; 220: 1636–1642. 12051502 10.2460/javma.2002.220.1636

[55] WHO Collaborating Centre for influenza at Centers for Disease Control and Prevention. CDC protocol of real-time RTPCR for influenza A(H1N1). Version 2009: Swine Influenza. Available: http://www.who.int/csr/resources/publications/swineflu/CDCRealtimeRTPCR_SwineH1Assay-2009_20090430.pdf

[56] Hole K, Velazques-Salinas L, Clavijo A. Improvement and optimization of a multiplex real-time reverse transcription polymerase chain reaction assay for the detection and typing of Vesicular stomatitis virus. J Vet Diagn Invest. 2010; 22: 428–433. 20453220 10.1177/104063871002200315

[57] Hakhverdyan M, Hagglun S, Larsen LE, Belák S. Evaluation of single-tube-fluorogenic RT-PCR assay for detection of bovine respiratory syncytial virus in clinical samples. J Virol Methods. 2005; 123:195–202. 15620402 10.1016/j.jviromet.2004.09.016 PMC7112851

[58] Reid SM, Ferris NP, Hutchings GH, King DP, Alexandersen S. Evaluation of real-time reverse transcription polymerase chain reaction assays for the detection of swine vesicular disease virus. J Virol Methods. 2004; 116: 169–176. 14738984 10.1016/j.jviromet.2003.11.007

[59] Avian Influenza EU Reference Laboratory, Animal and Plant Health Agency, UK. H7 Eurasian RealTime PCRs for the detection and pathotyping of Eurasian H7 avian influenza isolates. 2007; SOP VI.536 edition 4 11/04/07.

[60] Payungporn S, Chutinimitkul S, Chaisingh A, Damrongwantanapokin S, Buranathai C, Amonsin A, et al Single step multiplex real-time RT-PCR for H5N1 influenza A virus detection. J Virol Methods. 2006; 131: 143–147. 16183140 10.1016/j.jviromet.2005.08.004

[61] Callison SA, Hilt DA, Boynton TO, Sample BF, Robison R, Swayne DE, et al Development and evaluation of a real-time Taqman RT-PCR assay for the detection of infectious bronchitis virus from infected chickens. J Virol Methods. 2006; 138: 60–65. 16934878 10.1016/j.jviromet.2006.07.018 PMC7112890

