# Correction: MeltMan: Optimization, Evaluation, and Universal Application of a qPCR System Integrating the TaqMan qPCR and Melting Analysis into a Single Assay

**DOI:** 10.1371/journal.pone.0196444

**Published:** 2018-04-23

**Authors:** Alexander Nagy, Lenka Černíková, Eliška Vitásková, Vlastimil Křivda, Ádám Dán, Zuzana Dirbáková, Helena Jiřincová, Bohumír Procházka, Kamil Sedlák, Martina Havlíčková

In S1 File, the alignment shown in Profile no. 1A is incorrect. Please see the corrected S1 File here.

## Supporting information

S1 File MeltMan application examples. (PDF)
