# Supplementary material for: MeltMan: Optimization, Evaluation, and Universal Application of a qPCR System Integrating the TaqMan qPCR and Melting Analysis into a Single Assay
Source: PLoS One. 2016 Mar 31;11(3):e0151204. doi: 10.1371/journal.pone.0151204 (PMC4816343; doi:10.1371/journal.pone.0151204)
Supplement: S2 File — (PDF) [file pone.0151204.s002.pdf]

MeltMan: optimization, evaluation, and universal application of a qPCR system integrating the TaqMan qPCR and melting analysis into a single assay

Supporting Information 2.

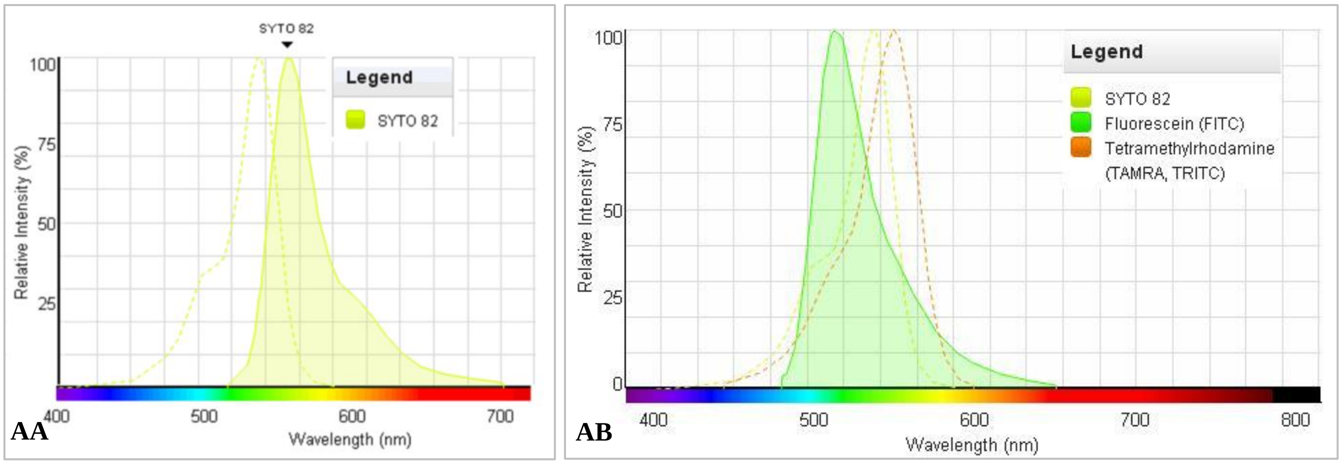

S2 Fig A. Fluorescence spectral overlap chart. (AA) the absorption (dashed) and emission spectra of the S82. (AB) the spectral overlap chart between the FAM emission (represented by fluorescein) and the S82 and TAMRA absorption spectra (dashed). The charts were generated by the Fluorescence Spectra Viewer (Life Technologies): http://www.lifetechnologies.com/cz/en/home/life-science/cell-analysis/labeling-chemistry/fluorescence-spectraviewer.html

S2 Table A. Reaction parameters of the S82 gradient. The mean data of three replicates are listed for the FMDV (brown) and IAV (blue) assays which are corresponding with Figs 1 and 2 in the manuscript and with the S2 Fig B.

[μM SYTO 82]	FAM					VIC	
	No. of points*	R^2	k	d(RFU) [%]	Cq±SD	Cq±SD	%H
**0**	6	0.999	1077.9	100	29.36±0.09	n.a.	n.a.
**1**	5	0.999	871.6	79.0	29.72±0.07	31.25±0.17	57.1
**2**	5	0.999	790.0	71.5	29.92±0.05	29.91±0.14	57.3
**3**	5	0.999	730.9	66.9	30.01±0.03	29.23±0.06	57.4
**4**	5	0.999	649.2	59.6	30.19±0.07	28.95±0.11	57.3
**5**	5	0.999	602.1	55.2	30.28±0.09	28.59±0.12	57.6
**6**	5	0.999	523.7	48.1	30.48±0.06	28.39±0.09	57.9
**7**	5	0.999	474.0	43,4	30.64±0.05	28.21±0.03	58.5
**8**	5	0.999	430.5	39.6	30.83±0.02	28.13±0.06	58.6
**9**	5	0.999	392.7	36.1	30.99±0.11	28.06±0.16	58.7
**10**	5	0.999	337.7	31.2	31.36±0.14	28.01±0.17	59.1

SYTO 82 [μM]	FAM					VIC	
	No. of points*	R^2	k	d(RFU) [%]	Cq±SD	Cq±SD	%H
**0**	7	0.999	1086.4	100	27.24±0.02	n.a	n.a
**1**	7	0.999	842.3	77.5	27.69±0.03	31.44±0.17	70.1
**2**	7	0.999	741.4	68.1	28.07±0.07	29.57±0.19	69.4
**3**	6	0.999	691.8	62.4	28.29±0.04	28.84±0.10	68.7
**4**	6	0.999	593.0	53.3	28.72±0.07	28.61±0.11	68.7
**5**	6	0.999	537.6	48.5	28.96±0.15	28.34±0.21	68.5
**6**	6	0.999	459.6	41.8	29.32±0.15	28.32±0.19	69.3
**7**	6	0.999	408.1	37.1	29.72±0.10	28.31±0.10	70.2
**8**	6	0.999	361.2	32.7	30.11±0.06	28.35±0.13	68.7
**9**	6	0.999	303.8	27.7	30.58±0.05	28.38±0.11	68.7
**10**	6	0.999	273.0	24.8	31.15±0.06	28.65±0.16	68.7

*Number of points used for the regression analysis of the exponential region.
k-slope values of the amplification curve.

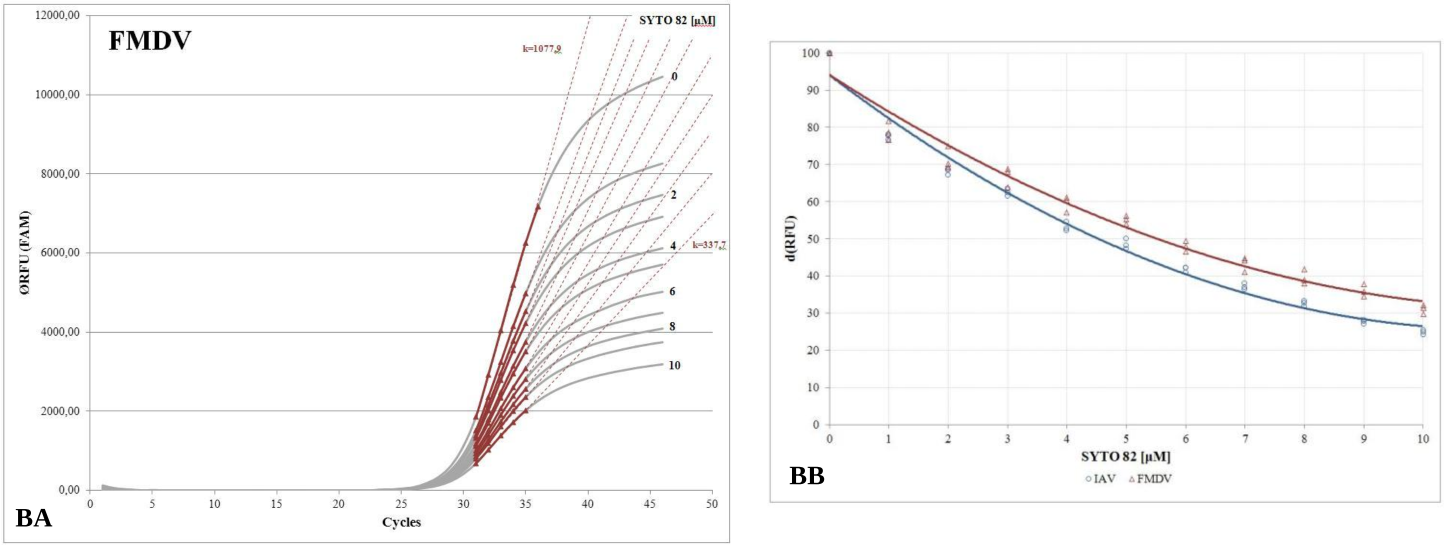

S2 Fig B. Effect of the S82 dye on the FAM probe fluorescence. (BA) the mean FAM d(RFU) data of the S82 gradient, ranges from 0 to 10µM, in 1µM increments, were shown for the FMDV assay (Table 1 in the manuscript) performed as a qPCR. Each curve represents an average of three TaqMan reaction replicates per S82 concentration for a fixed initial synthetic DNA standard amount of 1e4 copies/µl. The linear regression lines drawn across the exponential region of each amplification curve are dashed, and the region used in the regression line construction is highlighted with brown triangles. The slope values *k* were designated for the two S82 concentration extremities. For additional details, please refer to S2 Table A. (BB) the d(RFU) plot constructed by plotting the particular IAV (blue) and FMDV (brown) d(RFU) FAM values in percentages against the S82 concentration.

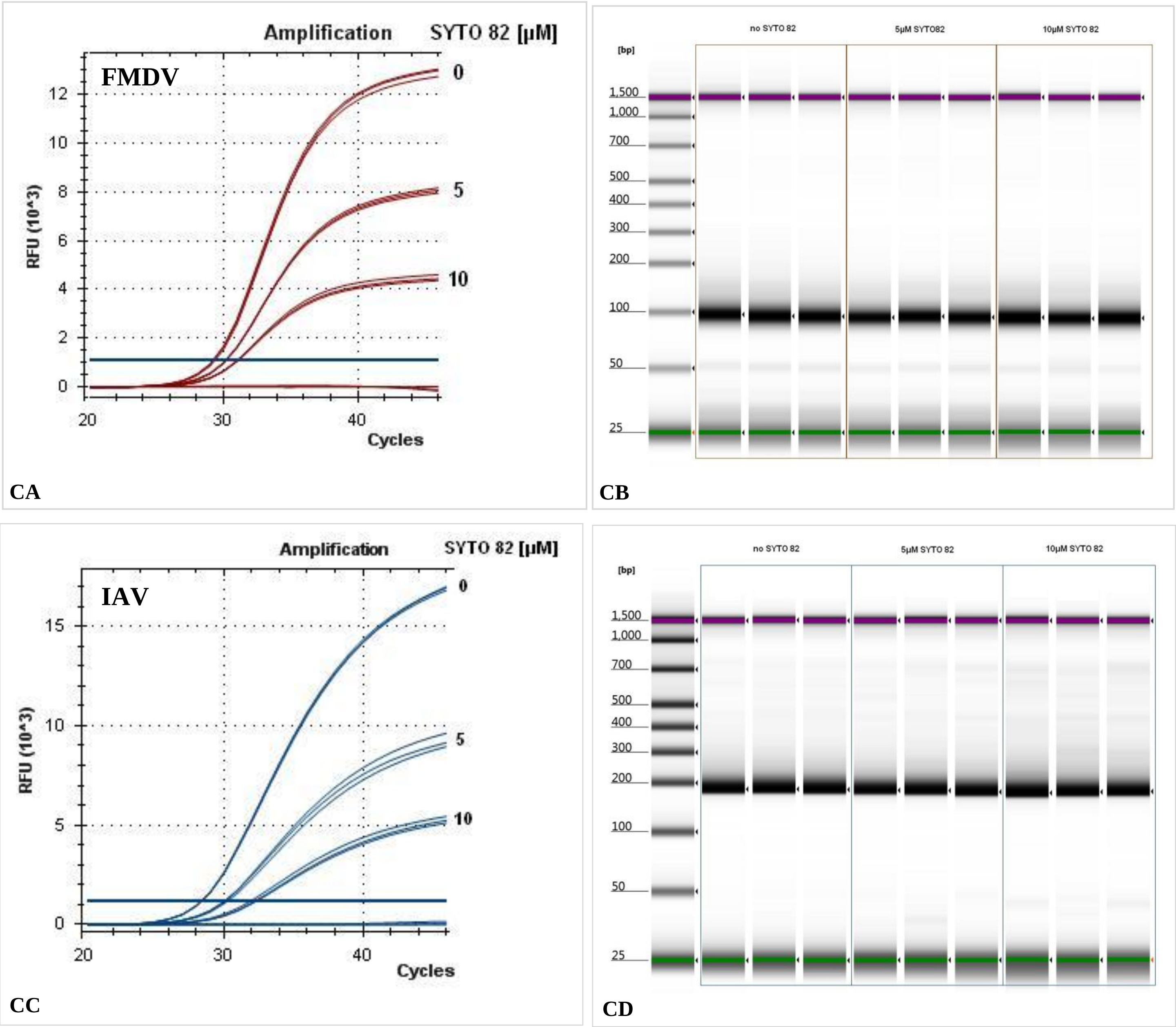

S2 Fig C. Electrophoretic analysis of the S82 gradient. The reaction products of three S82 concentration points 0, 5 and 10 µM of the FMDV (brown) and IAV (blue) assays performed as qPCR and amplifying a fixed initial template amount of 1e4 copies/µl (CA and CC) were subjected to electrophoresis (CB, and CD; Tape Station 2200, Agilent Technologies). The comparison of the amplicon quantities estimated by the instrument was shown in S2 Table B.

S2 Table B. Comparison the of the reaction product quantities at specific S82 concentration points.

FMDV				IAV			
**SYTO 82 [µM]**	**1. [ng/µl]**	**2. [ng/µl]**	**3. [ng/µl]**	**SYTO 82 [µM]**	**1. [ng/µl]**	**2. [ng/µl]**	**3. [ng/µl]**
**0**	15.1	16.2	16.6	**0**	11.2	11.3	11.5
**5**	14.1	16.1	14.5	**5**	10.0	10.9	11.5
**10**	18.8	15.2	18.2	**10**	12.3	11.6	11.6

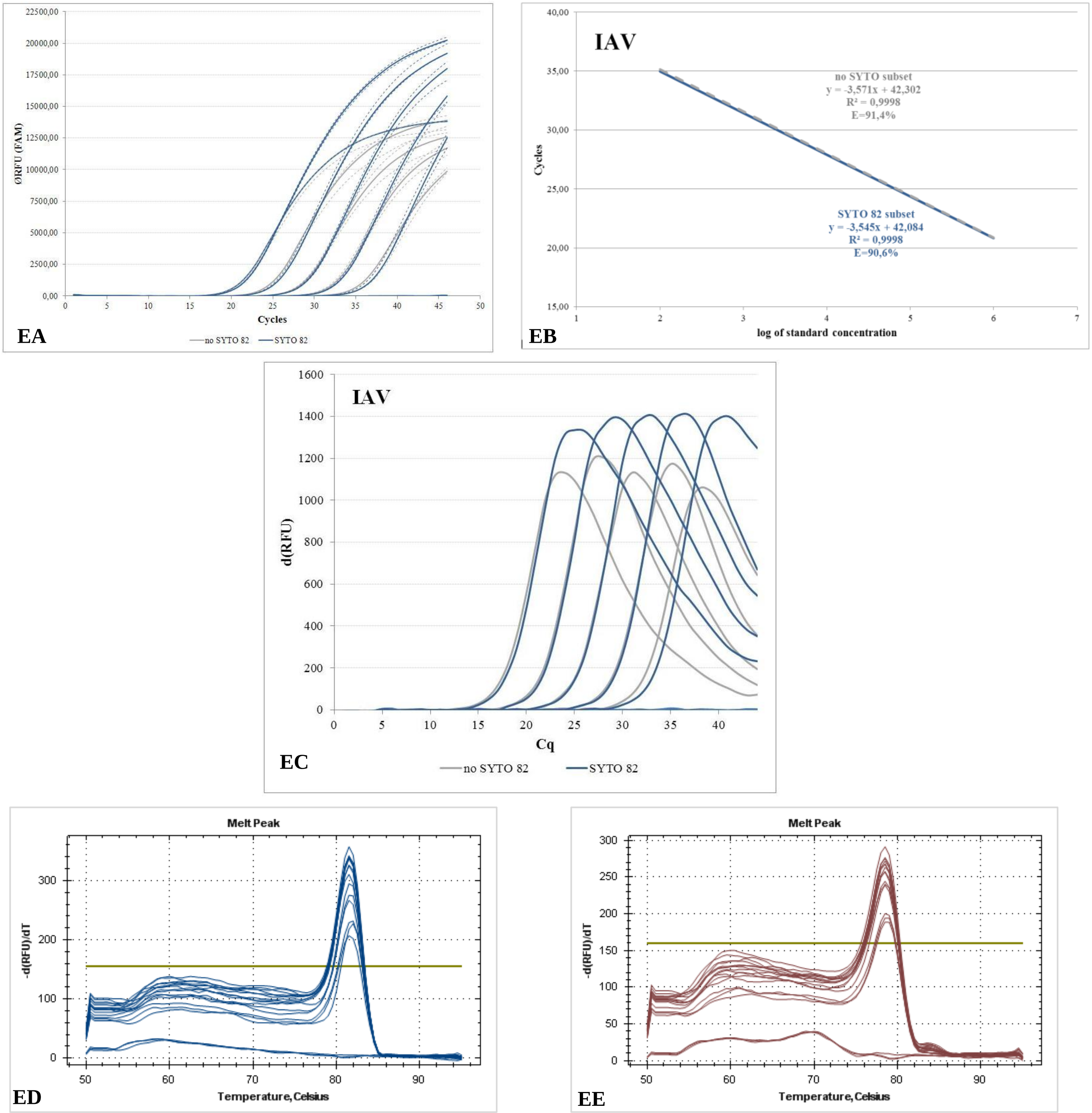

S2 Fig E. Evaluation of the MeltMan reaction. (EA, EB) the amplification and calibration curves of the IAV qPCR assay from 1e2 to 1e6 of the synthetic DNA template copies/µl. For clarity certain curves were visualized in dashed representation. (EC) represents the first derivative of the FAM amplification curve per one replicate series of the IAV assay. The no S82 subset was highlighted in grey and the S82 in blue, respectively. (ED, EE) represent the melting peaks of the IAV and FMDV (brown) assays evaluated in the VIC channel. The data are corresponding with S2 Table C.

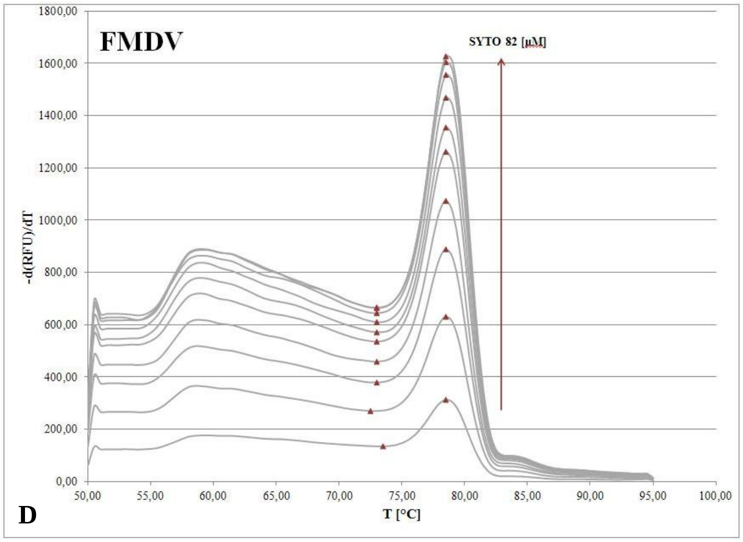

S2 Fig D. Effect of the SYTO 82 on the melting peak profiles. The melt curve derivative results of the FMDV qPCR assay gathered in the VIC channel. Each melting peak represents an average of three TaqMan reaction replicates per S82 concentration with a fixed initial template amount of 1e4 copies/µl. The S82 gradient, ranges from 0 to 10µM, in 1µM increments, was designated with a brown arrow. For each peak, the left tail local minimum and maximum values used for peak height H and proportionality, %H, estimation were designated with brown triangles. The data are corresponding with S2 Table B.

S2 Table C. Comparison of the reaction parameters between the no S82 and S82 reactions. The mean data of three replicates are listed for the FMDV (brown) and IAV (blue) assays and are corresponding with Fig 3 in the manuscript and with the S2 Figs D and E.

FMDV											
Standard conc.	no SYTO 82				SYTO 82						
	FAM				FAM					VIC	
	No. of points*	Cq±SD	R^2	k	No. of points*	ØCq±SD	R^2	k	d(RFU) [%]	Cq±SD	%H
**1e2**	7	35.01±0.09	0.999	997.9	7	34.86±0.12	0.999	1356.2	23.6	33.46±0.12	59.7
**1e3**	6	31.30±0.62	0.998	1090.5	7	31.26±0.11	0.999	1435.5	23.3	29.92±0.06	59.8
**1e4**	6	27.50±0.14	0.999	1088.7	7	27.45±0.09	0.999	1436.1	23.5	26.10±0.10	58.8
**1e5**	6	24.04±0.12	0.999	1140.7	7	23.96±0.09	0.999	1458.3	21.2	22.57±0.22	59.2
**1e6**	6	20.45±0.09	0.999	1110.9	7	20.38±0.11	0.999	1471.3	24.0	19.12±0.07	58.5

IAV											
Standard conc.	no SYTO 82				SYTO 82						
	FAM				FAM					VIC	
	No. of points*	Cq±SD	R^2	k	No. of points*	Cq±SD	R^2	k	d(RFU) [%]	Cq±SD	%H
**1e2**	7	35.26±0.12	0.999	1038.2	8	35.07±0.17	0.999	1297.9	18.8	34.56±0.11	72.9
**1e3**	6	31.37±0.09	0.999	1092.0	8	31.49±0.06	0.999	1298.8	16.3	30.84±0.09	70.5
**1e4**	7	27.91±0.11	0.999	1052.8	9	27.99±0.05	0.999	1287.0	17.5	27.35±0.05	70.4
**1e5**	7	24.28±0.04	0.999	1089.4	8	24.40±0.08	0.999	1309.3	15.9	23.92±0.10	69.5
**1e6**	7	20.89±0.13	0.999	1057.4	8	20.95±0.04	0.999	1274.7	16.5	20.37±0.07	69.2

*Number of points used for the regression analysis of the exponential region
k-slope values of the amplification curve

S2 Table D. Comparison of the sensitivity between the no S82 and S82 reactions. The mean data of ten replicates are listed for the qPCR and RT-qPCR assays and are corresponding with Fig 4 in the manuscript and with S2 Fig F. For the abbreviations please refer to Table 1 in the manuscript.

Method	Amplicon length	Specimen	no SYTO 82	SYTO 82		
			FAM	FAM		VIC
			Cq±SD	Cq±SD	d(RFU) [%]	H±SD [%]
EHV-1	60	equine org. susp.	33.91±0.20	33.43±0.14	33.7±2.9	72.9±2.7
Chicken DNA	76	chicken meat	32.66±0.16	32.28±0.16	26.4±4.5	70.7±1.3
TBEV	98	goat milk	34.75±0.27	34.95±0.22	27.9±8.7	50.6±2.1
Celery DNA	101	pork pudding	30.43±0.32	30.96±0.08	20.5±4.9	70.7±0.5
OvHv-2	131	bovine blood	30.78±0.08	30.37±0.21	20.5±3,8	73.6±11
IAV	182	duck cl. swab	33.24±0.39	33.17±0.19	22.8±9,0	77.3±2.8
EAV	204	equine semen	31.64±0.14	31.52±0.18	-3,4±15	68.6±1.1

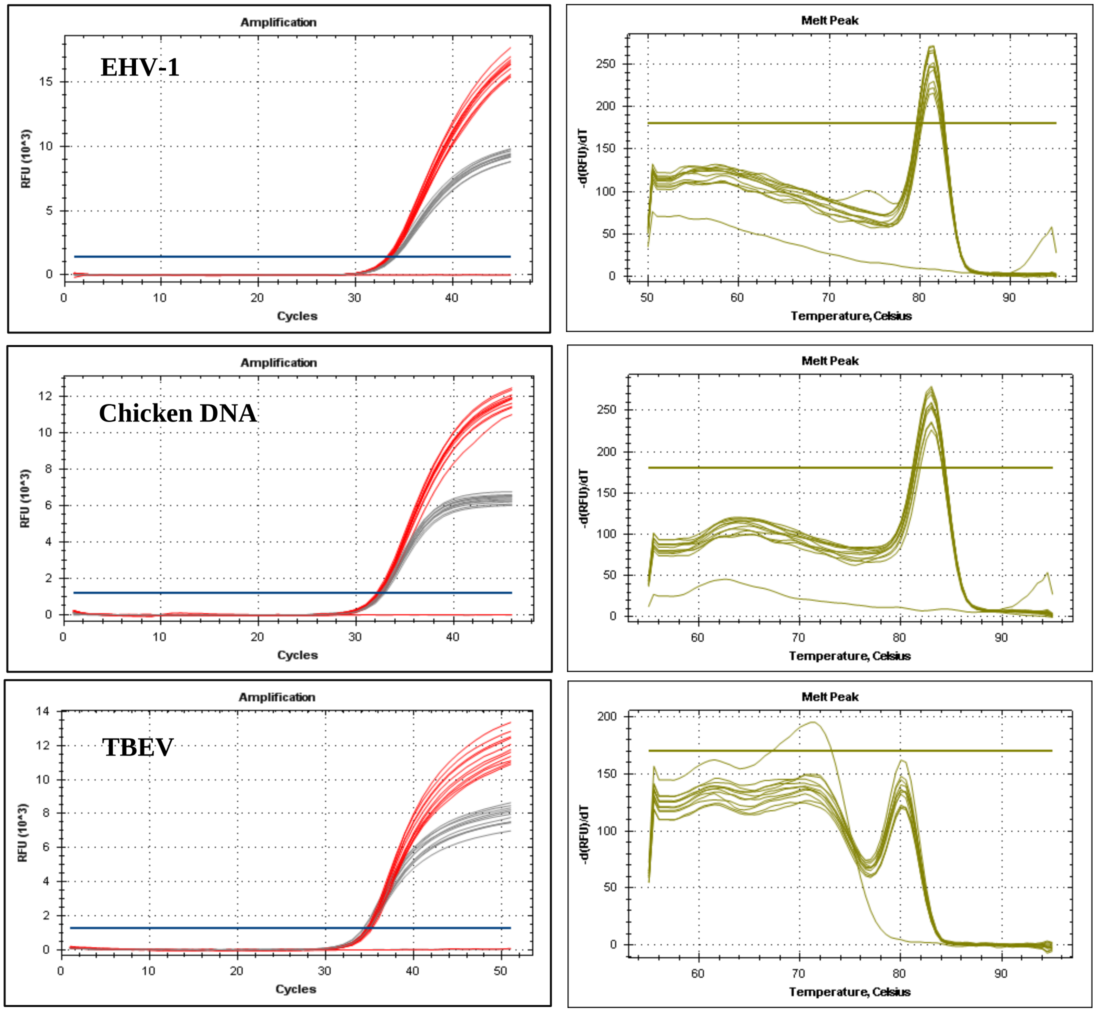

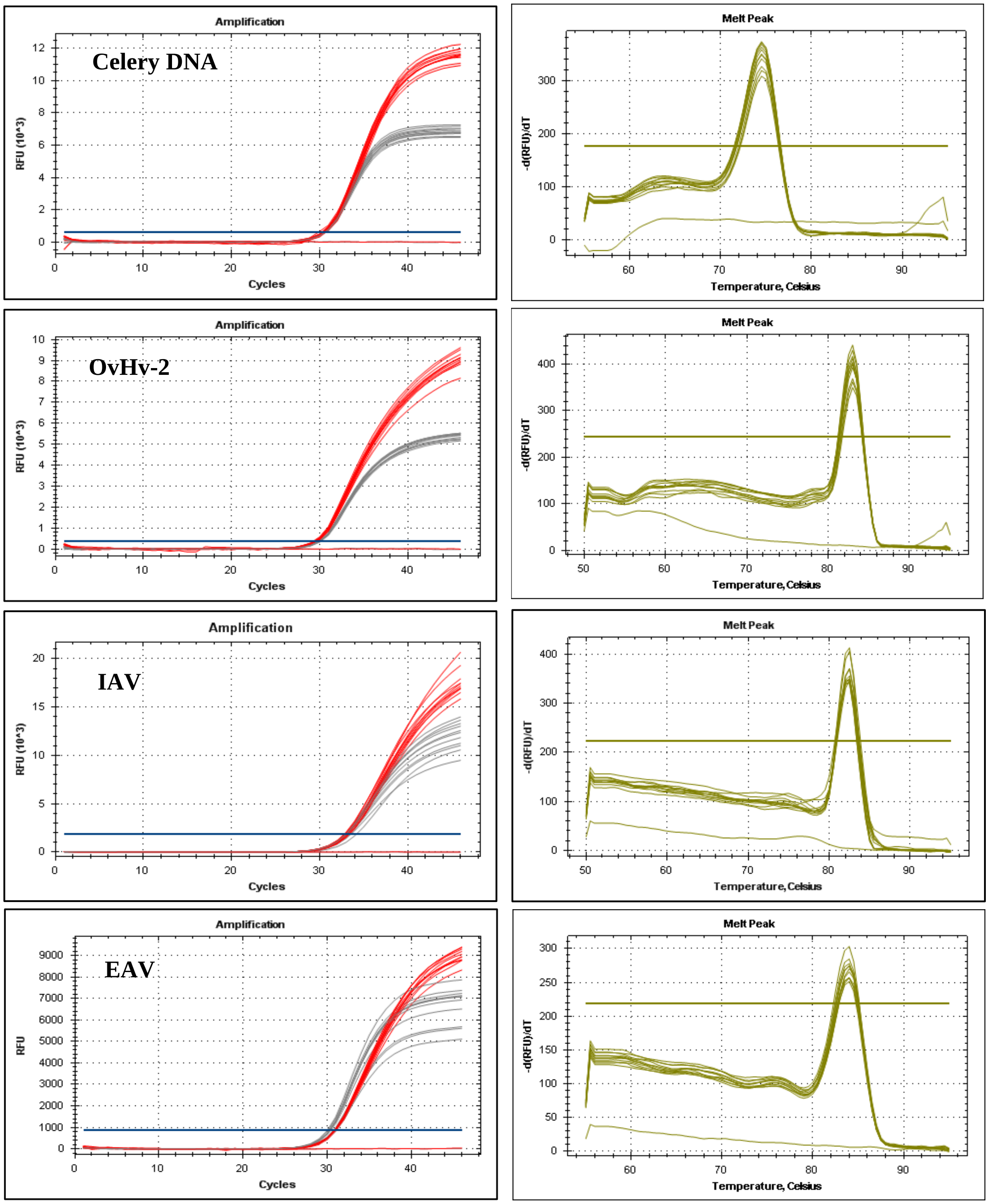

S2 Fig F. Sensitivity of the MeltMan reaction system. Seven TaqMan assays: EHV-1, Chicken DNA, TBEV, Celery DNA, OvHV-2, IAV, and EAV (manuscript Table 1) were analysed by using diluted field specimens to reach Cq values greater than 30. The assays were prepared in two subsets no S82 (grey) and S82 (red) with ten replicates/ subset. The figure represents the amplification (left) and melting profiles (right) for each particular assay gathered in the FAM and VIC channels respectively. The data are corresponding with S2 Table D.

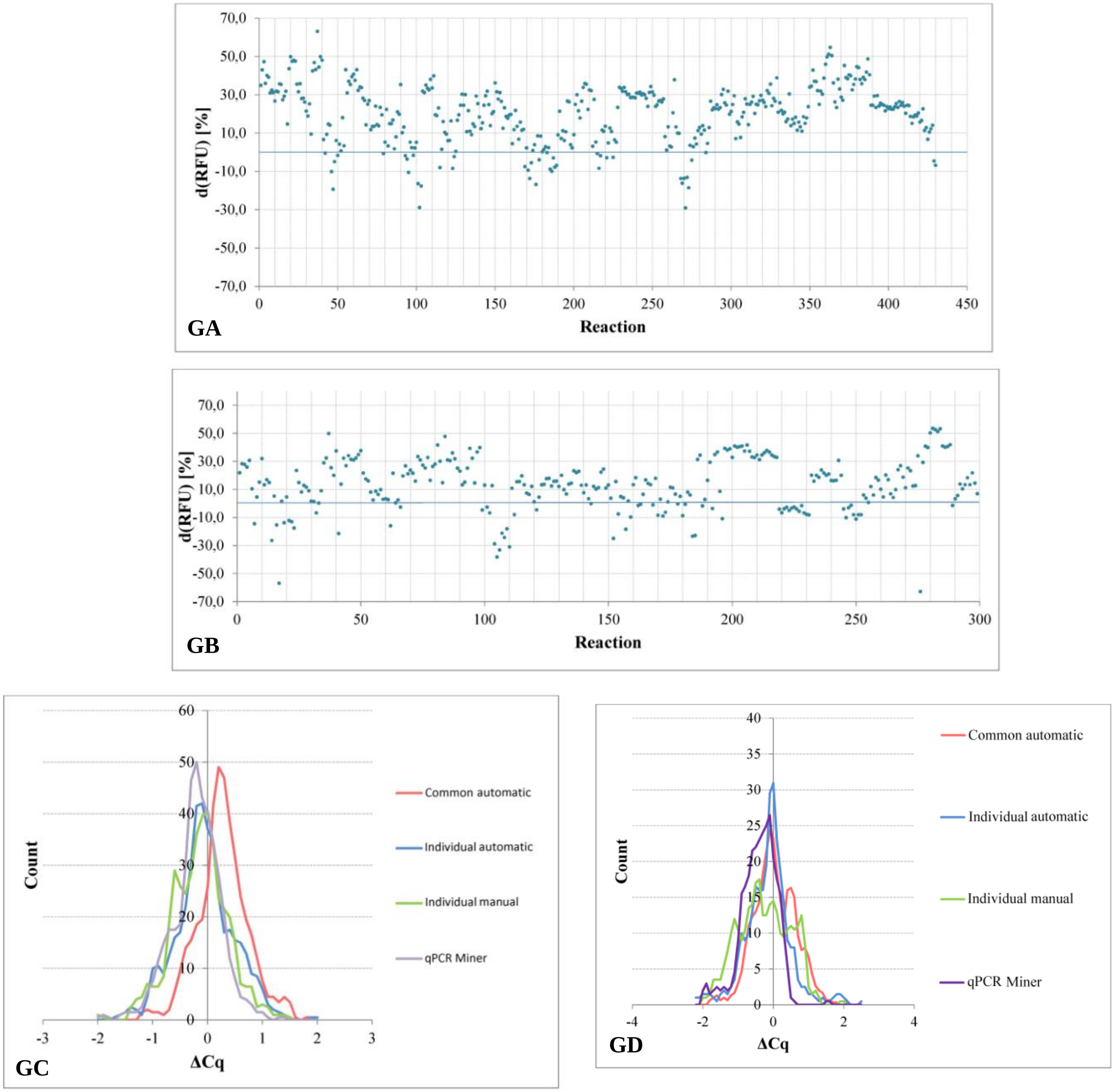

S2 Fig G. The d(RFU) FAM scatter plots, and ΔCq distribution of the DNA (Figs GA, GC) and RNA (Figs GB, GD) sample pools. (GA, GB) the d(RFU) FAM scatter plots summarize the differences in FAM curve fluorescence of the S82 reactions relative to the no S82 counterparts. (GC, GD) illustrate the distribution of the particular ΔCq values per each baseline strategy used.
