# Supplementary material for: Correction: MeltMan: Optimization, Evaluation, and Universal Application of a qPCR System Integrating the TaqMan qPCR and Melting Analysis into a Single Assay
Source: PLoS One. 2018 Apr 23;13(4):e0196444. doi: 10.1371/journal.pone.0196444 (PMC5912733; doi:10.1371/journal.pone.0196444)
Supplement: S1 File — (PDF) [file pone.0196444.s001.pdf]

MeltMan: optimization, evaluation, and universal application of a qPCR system integrating the TaqMan qPCR and melting analysis into a single assay

Supporting Information 1.

Application examples of the MeltMan assay:

1. Probe binding anomalies
2. FAM curve "tails"
3. Weird FAM curves
4. Invisible byproducts
5. Dye relocation and peak discernibility

Profile no. 1A: Probe binding failure

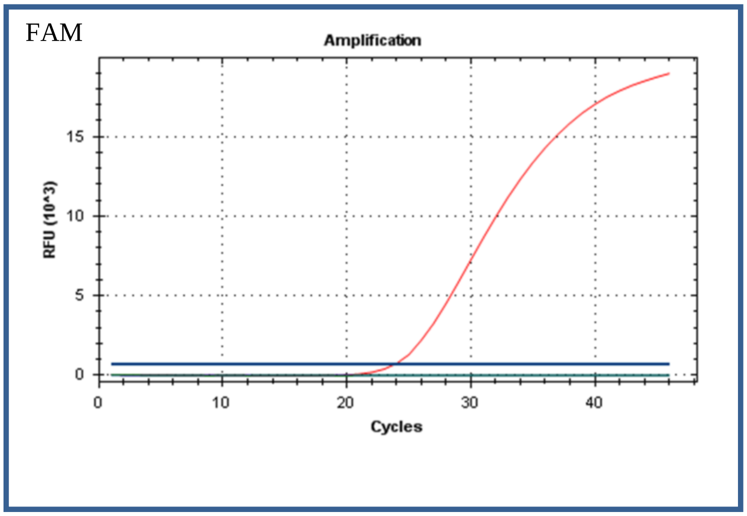

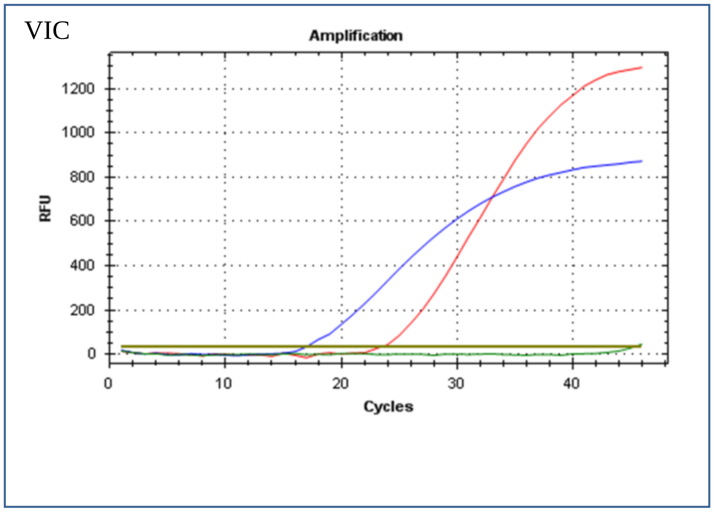

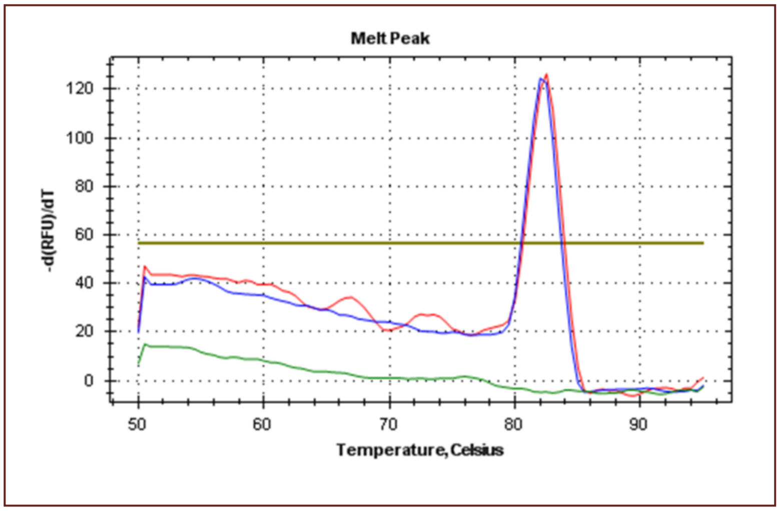

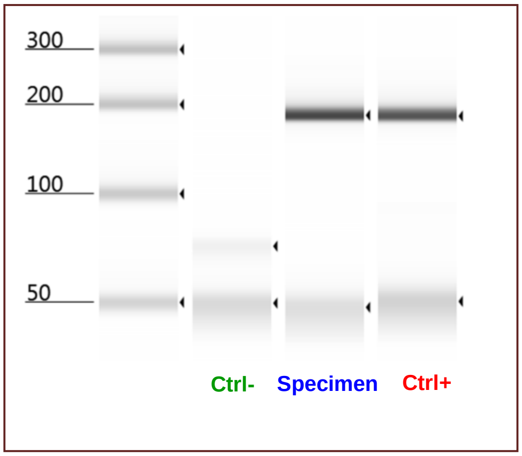

Analysis	Influenza A Virus, IAV
ID	**126/12**
Specimen	A/Czech Republic/126/2012 (H3N2), MDCK culture
Method/Gene	Nagy et al., 2010; *Arch. Virol.*, **155**, 665-673/ M segment
Amplicon length	182 bp
NA extraction	MagNAPure Compact Total NA Isolation Kit (Roche)
qPCR Kit/platform	QuantiTect Probe RT-PCR Kit (Qiagen)/CFX96
Electrophoresis	D1000 ScreenTape, TapeStation 2200, (Agilent Technologies)
Sequencing	BDTv3.1, GA 3130 (Life Technologies)

```
          ....|...
ctrl+     GTGCCCAG
126/12    .....A..
```

Notes: The TaqMan assay evaluated in the FAM channel shows amplification curve only for the positive control (red) and absence of the amplicon specific signal both in the negative control (green) and the specimen (blue). Hence, the assay suggests clear negativity.

However, performing the assay in the MeltMan format also revealed amplification in the specimen vial detected in the VIC channel. Subsequent melting analysis identified a single peak which tightly overlapped with the positive control. Similarly, the electrophoresis revealed a single band with expected amplicon length of 182bp. Hence, the analyzed specimen was evidently positive and the initial TaqMan assay result was false negative.

To decipher this observation sequencing of the M segment was performed (GenBank JX913062) which identified a single G/A transition in the probe binding region. Since the probe used did not tolerate any mismatches in the targeted sequence (Nagy et al., 2010) the G/A substitution evidently abolished the probe binding and led to false negativity despite the high viral burden. This example is the extremity of the profile 1C.

Profile no. 1B: Probe binding failure

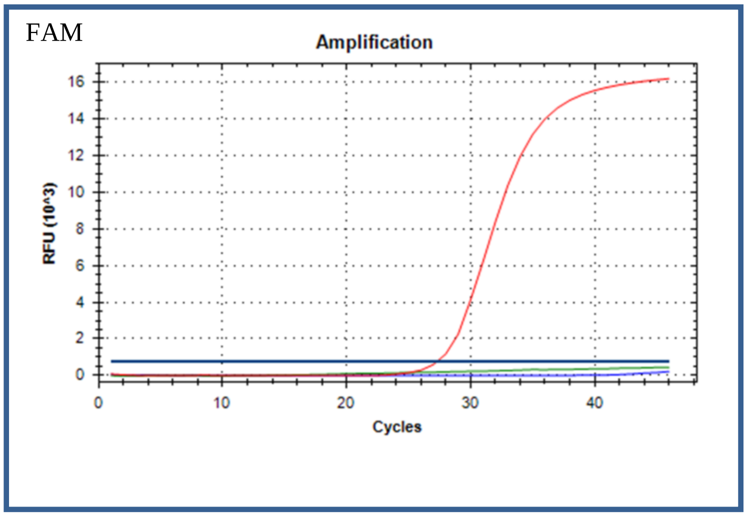

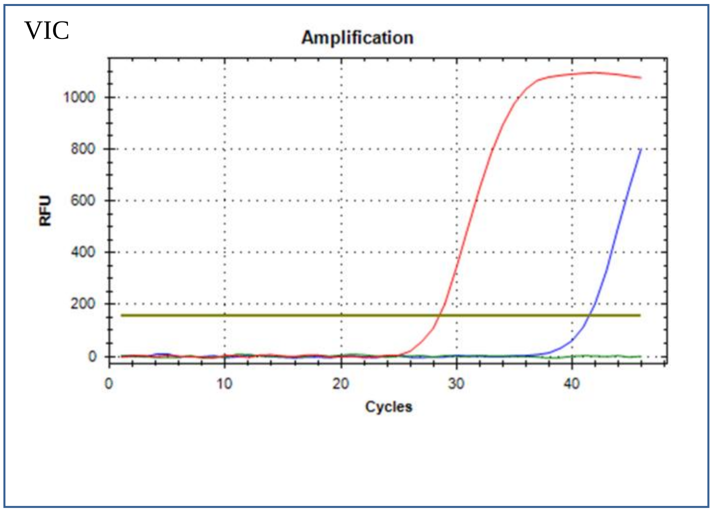

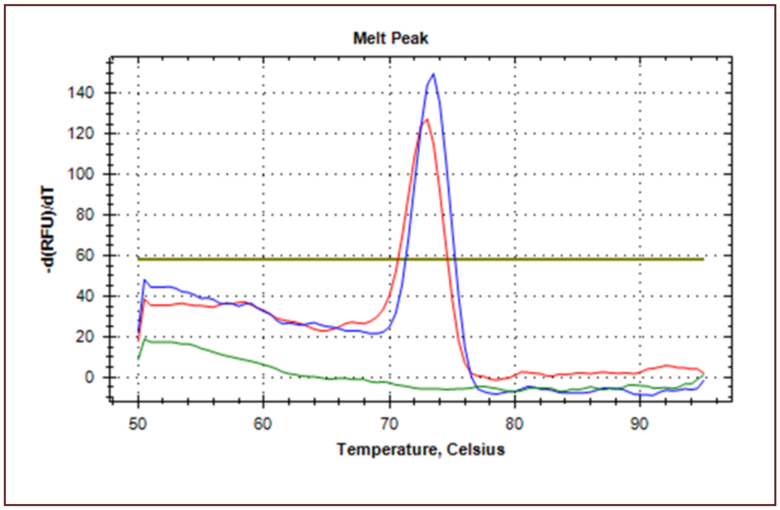

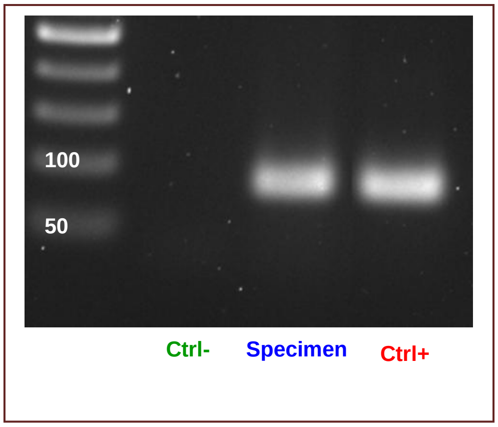

**Analysis**	**Felid Parvovirus, FPV**
ID	6736/15
Specimen	**organ suspension**
Method/Gene	Decaro et al 2008, *J. Virol. Methods,* **147**, 67-71/ VP2
Amplicon length	93bp
NA extraction	MagNAPure Compact Total NA Isolation Kit (Roche)
qPCR Kit/platform	QuantiTect Probe PCR Kit (Qiagen)/CFX96
Electrophoresis	2% Agarose gel, TAE buffer, 150V
Sequencing	BDTv3.1, GA 3130 (Life Technologies)

```
                     10        20
            ....|....|....|....|....|....
6736/15     ATGGGaAATGcaaaCTATATTaCtgaAgC
FPV Probe   .........A..G.......
CPV Probe   .........A..........
```

Notes: The TaqMan assay for a specimen with FPV detection inquiry shows negativity in the FAM channel with a signal observed only in the positive control (red).

Nevertheless, the MeltMan assay revealed amplification also in the specimen vial (blue) registered in the yellow channel. The melting analysis indicated a single peak which tightly overlapped with the positive control. The electrophoresis again confirmed a single band with expected amplicon length of 93bp.

Amplicon sequencing and subsequent BLAST analysis suggested that the observed fragment was not of FPV but in fact CPV-2 (canid parvovirus) origin. Originally, the Decaro et al., 2008 TaqMan assay employs one primer pair for both FPV and CPV-2 detection while the discrimination occurs via specific MGB probes. Hence, albeit the common primers generated the CPV-2 amplicon it was undetected due to FPV probe binding failure and was visible only upon implementing the MeltMan approach. Interestingly, the alignment revealed sequence differences between the CPV-2 amplicon and the CPV-2 specific probe as well which strongly suggests that even using the right probe will still probably lead to a weird curve due to weak probe binding (Profile 1C).

Profile no. 1C: Flat FAM curves – weak probe binding

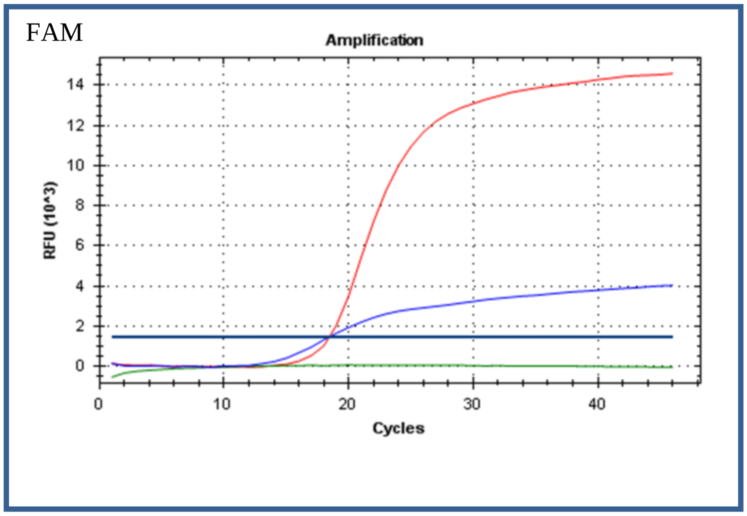

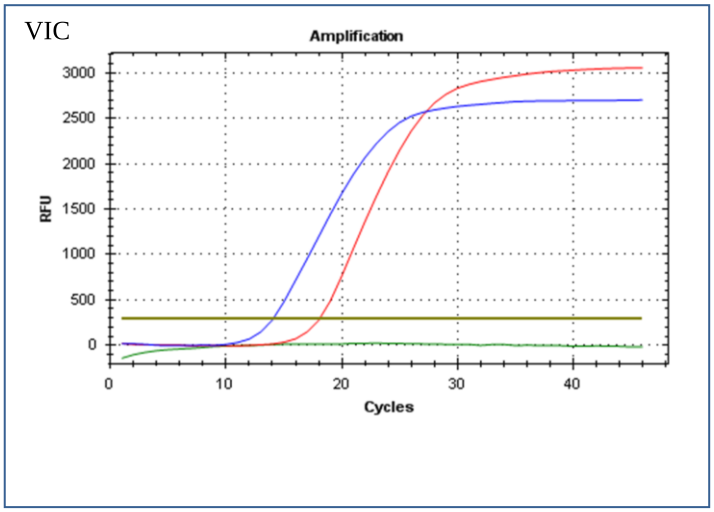

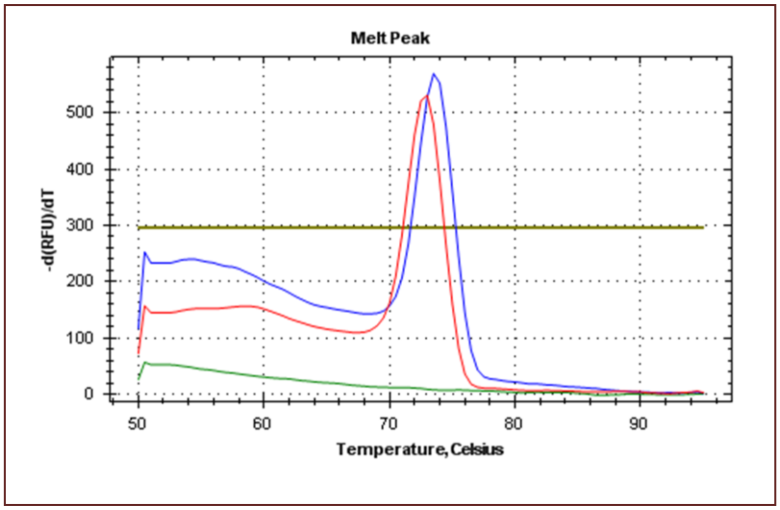

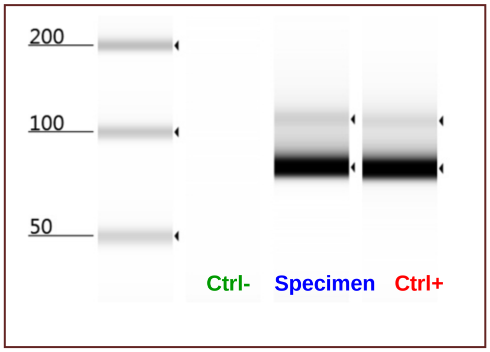

Analysis	Canid Parvovirus, CPV-2
ID	12364/09
Specimen	**dog-organ suspension**
Method/Gene	Decaro et al 2008, *J. Virol. Methods*, **147**, 67-71/ VP2
Amplicon length	93bp
NA extraction	MagNAPure Compact Total NA Isolation Kit (Roche)
qPCR Kit/platform	QuantiTect Probe PCR Kit (Qiagen)/CFX96
Electrophoresis	D1000 ScreenTape, TapeStation 2200 (Agilent Technologies)
Sequencing	BDTv3.1, GA 3130 (Life Technologies)

```
                      10        20
              ....|....|....|....|
Probe         ATGGGAAATACAAACtATAT
12364/09      .........G..........
```

Notes: The CPV-2 TaqMan assay shows a steep FAM curve for the positive control (red), but a flat curve for the specimen (blue). Is the specimen CPV-2 positive, or negative?

When the assay was performed in the MeltMan format the specimen showed a clear amplification in the VIC channel. Subsequent melting analysis revealed a single melting peak with only +0.5°C difference from the positive control. This suggests positivity with high confidence and was supported by electrophoresis of the reaction products. Hence, the analyzed specimen was evidently MeltMan positive while the initial TaqMan assay was inconclusive.

To refine this observation, the amplicon was subjected to sequence and BLAST analysis which proved the CPV-2 virus. In addition, one G/A transition situated in the middle of the probe binding region, which evidently weaken the TaqMan MGB probe hybridization, was identified. Hence, the MeltMan analysis showed clear positivity while the initial TaqMan assay was inconclusive with further analyses required to elucidate the result obtained. A second mutation in the probe binding region will led to false negativity with a high probability (Profile 1B).

Profile no. 1D: Flat FAM curves – weak probe binding

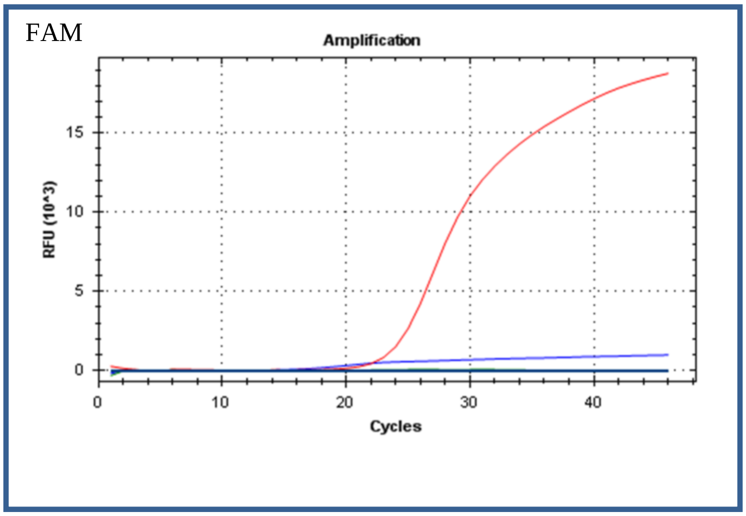

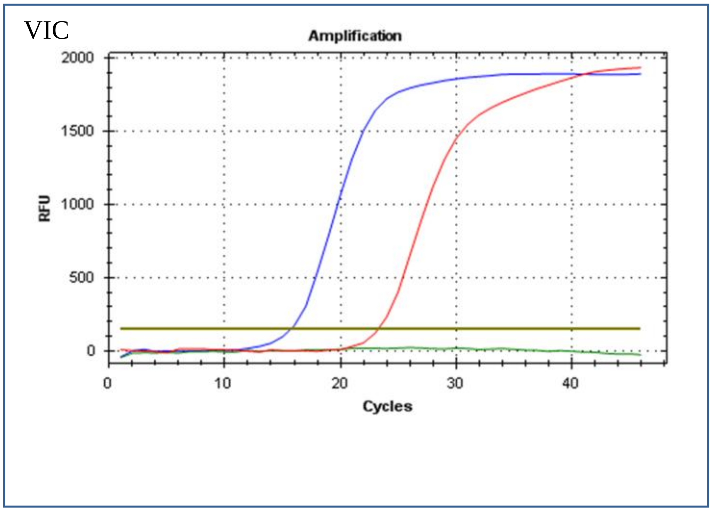

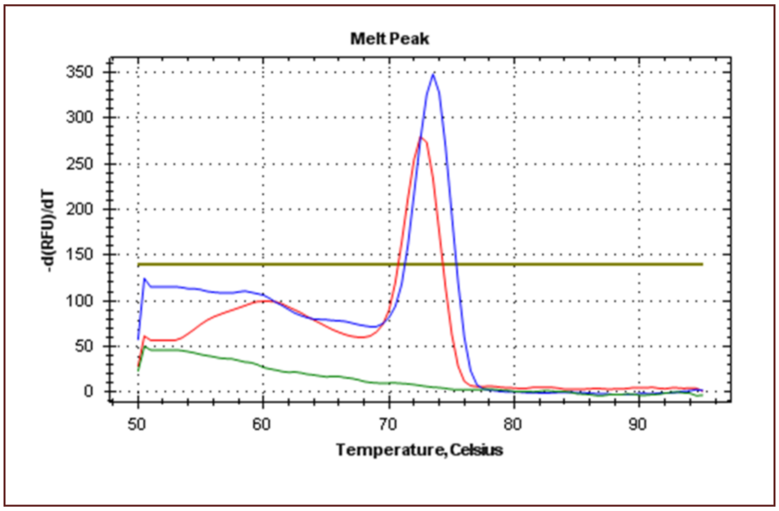

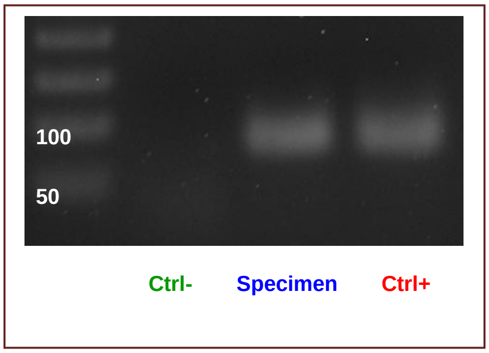

Analysis	Canid Parvovirus, CPV-2
ID	14823/15
Specimen	**dog-organ suspension**
Method/Gene	Decaro et al 2008, *J. Virol. Methods*, **147**, 67-71/ VP2
Amplicon length	93bp
NA extraction	MagNAPure Compact Total NA Isolation Kit (Roche)
qPCR Kit/platform	QuantiTect Probe PCR Kit (Qiagen)/CFX96
Electrophoresis	2% Agarose gel, TAE buffer, 150V
Sequencing	BDTv3.1, GA 3130 (Life Technologies)

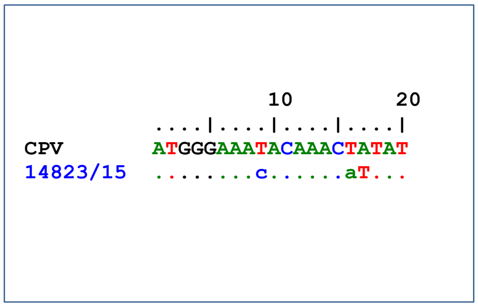

Notes: The CPV-2 TaqMan assay shows negativity for the specimen.

When the assay was performed in the MeltMan format a clear amplification in the VIC channel was observed. Subsequent melting analysis revealed a single melting peak with only +1°C difference from the positive control suggesting positivity with high confidence and was supported by electrophoresis of the reaction products. Hence, the analyzed specimen was evidently MeltMan positive while the initial TaqMan assay was false negative.

To refine this observation, the amplicon was subjected to sequence analysis which revealed three mutations spanning within the probe binding region abolishing the TaqMan MGB probe hybridization. BLAST analysis identified a rare CPV-2c genotype which was further confirmed by additional assays.

Profile no. 2A: Simple FAM curve “tails”

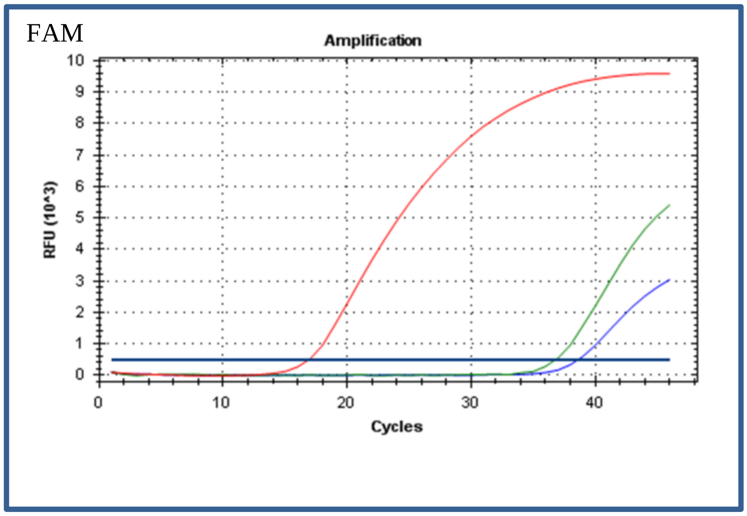

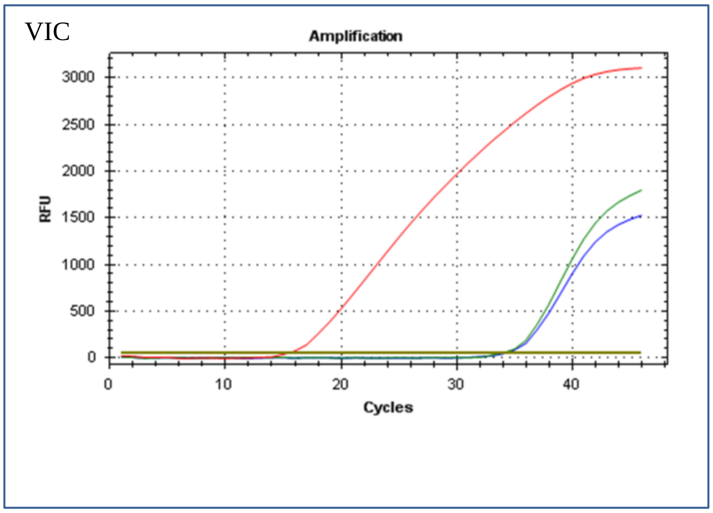

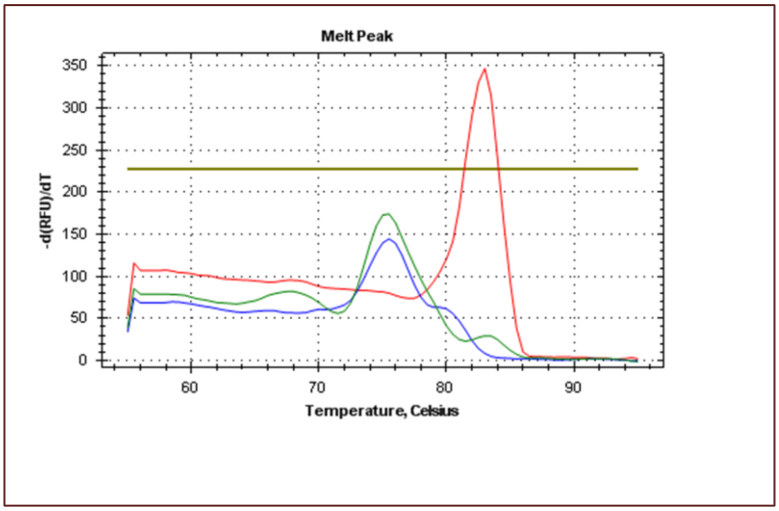

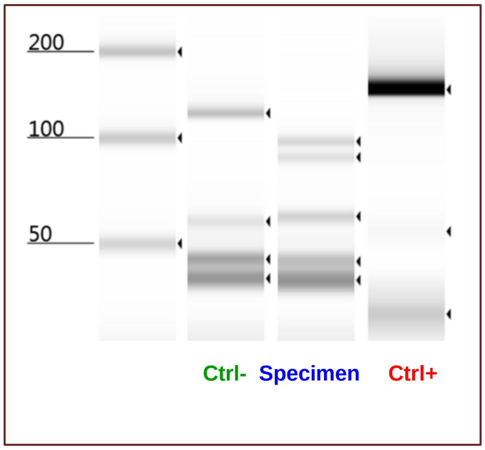

**Analysis**	**Bovine Viral Diarrhea Virus 1, BVDV1**
ID	9855/14
Specimen	**Bovine blood**
Method/Gene	Lettelier et al., 2003; *J. Virol. Methods,* **114**, 21-27/ 5'UTR
Amplicon length	168bp
NA extraction	MagNAPure Compact Total NA Isolation Kit (Roche)
qPCR Kit/platform	QuantiTect Probe RT-PCR Kit (Qiagen)/CFX96
Electrophoresis	D1000 ScreenTape, TapeStation 2200, (Agilent Technologies)

Notes: The TaqMan assay shows weak positivity in the FAM channel both for the specimen (blue) and the negative control (green) with Cq values of 38.61 and 36.80 respectively. The interpretation of such “tails” with Cq ≥36 is always problematic and they may also indicate contamination.

However, obtaining such result in the MeltMan format allows immediate clarification of this FAM curves via melting analysis which revealed that both of the specimen and negative control signals resulted from nonspecific probe hydrolysis or degradation and not from contamination. For completeness the electrophoretic pattern is also attached.

Profile no. 2B: Ambiguous FAM curve “tails”

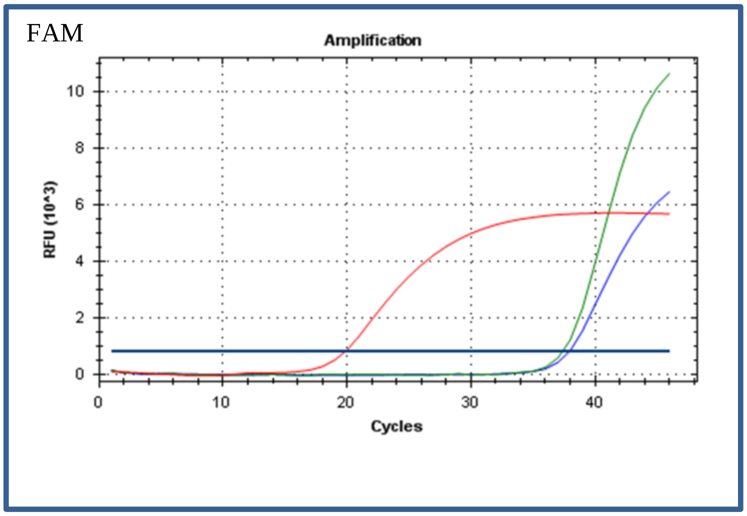

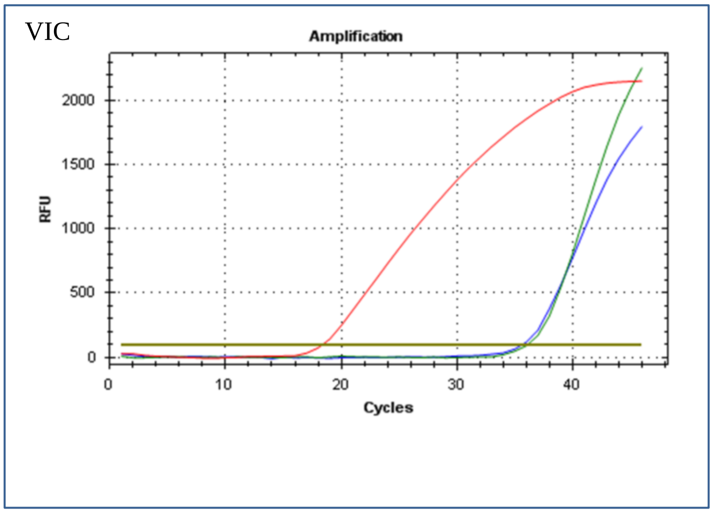

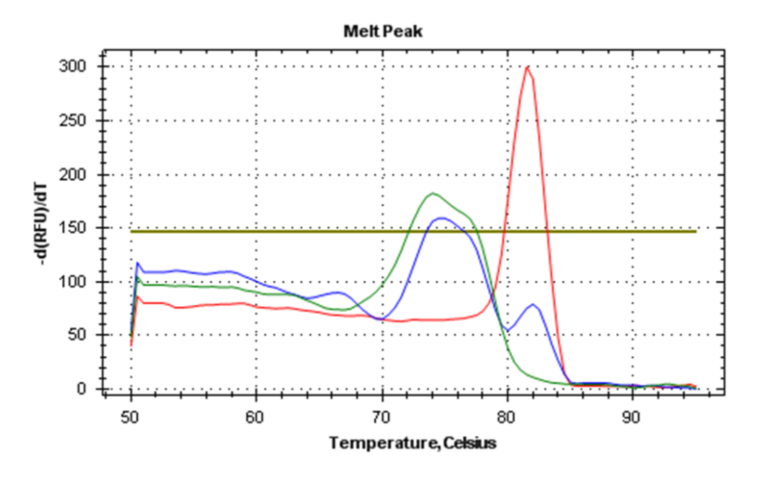

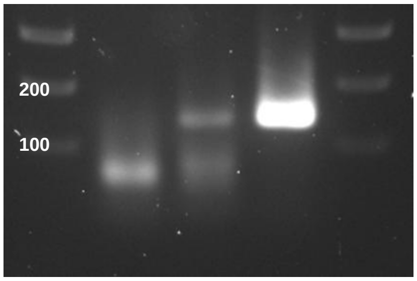

Ctrl- Specimen Ctrl+

```
                   10        20        30        40        50        60        70        80        90       100
               ....|....|....|....|....|....|....|....|....|....|....|....|....|....|....|....|....|....|....|....|...
IBV CTRL+      GGGCTACGTTCTCGCATAAGGTCGGCTATACGACGTTTGTAGGGGGTAGTGCCaAaCAACCCCTGAGGTGacAGGTTCTGGTGGTGTTTAGTGAGCAGACATA
9738/14        ................C........T......G......................................................................
probe                                                                          .........T............
```

Analysis	Infectious Bronchitis Virus, IBV
ID	9738/14
Specimen	**Pooled chicken organ suspension**
Method/Gene	Callison et al. 2006; *J. Virol. Methods,* **138**, 60-65/ 5’UTR
Amplicon length	143bp
NA extraction	MagNAPure Compact Total NA Isolation Kit (Roche)
qPCR Kit/platform	QuantiTect Probe RT-PCR Kit (Qiagen)/CFX96
Electrophoresis	2% Agarose gel, TAE buffer, 150V
Sequencing	BDTv3.1, GA 3130 (Life Technologies)

Notes: The TaqMan assay exhibits FAM curve tails both for the specimen (blue) and the negative control (green) with Cq of 37.92 and 37.35 respectively. This resembles the profile 2A and indicates inconclusive results and probably contamination.

Reanalysis in the MeltMan format showed similar pattern in the VIC channel. However, a subsequent melting analysis did not show specific amplicon in the negative control which suggests that the FAM signal observed was nonspecific. On the contrary, the melting analysis further revealed a specific peak in the specimen with only 0.5°C shift in Tm in comparison with the positive control (red). Similarly, the electrophoresis confirmed a weak but specific band.

Sequencing and BLAST analysis proved the IBV virus sequence. In addition, the sequence alignment revealed three nucleotide differences relative to the positive control. Interestingly, both sequences held one T/G transversion in the probe binding region.

Profile no. 3A: Nonspecific probe hydrolysis or disintegration

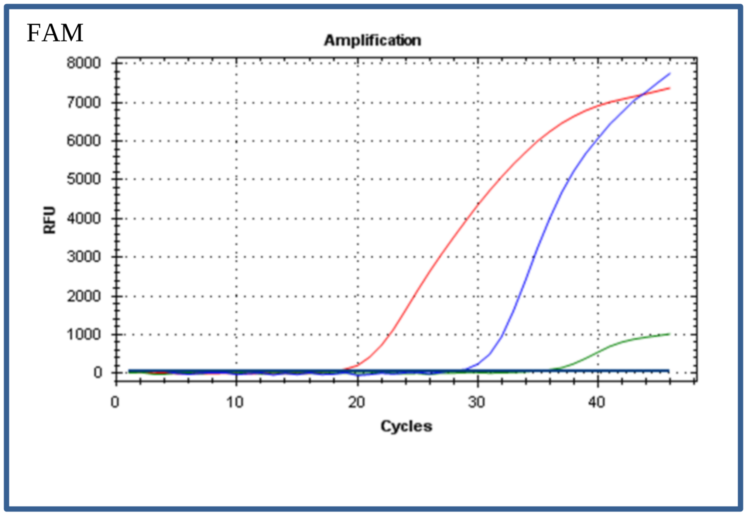

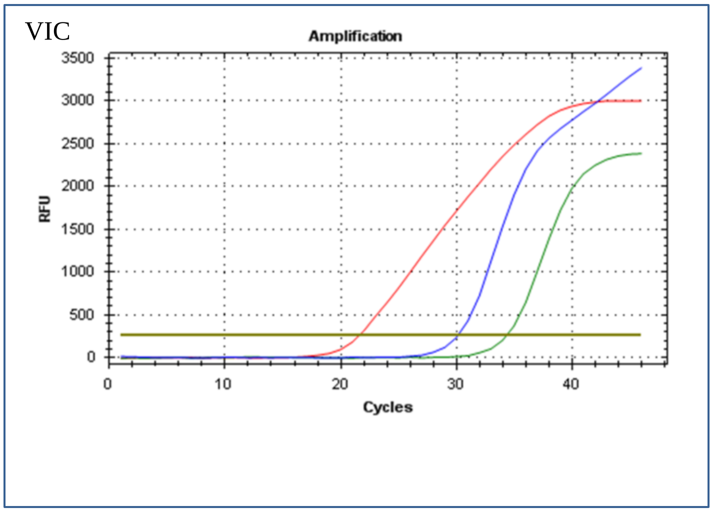

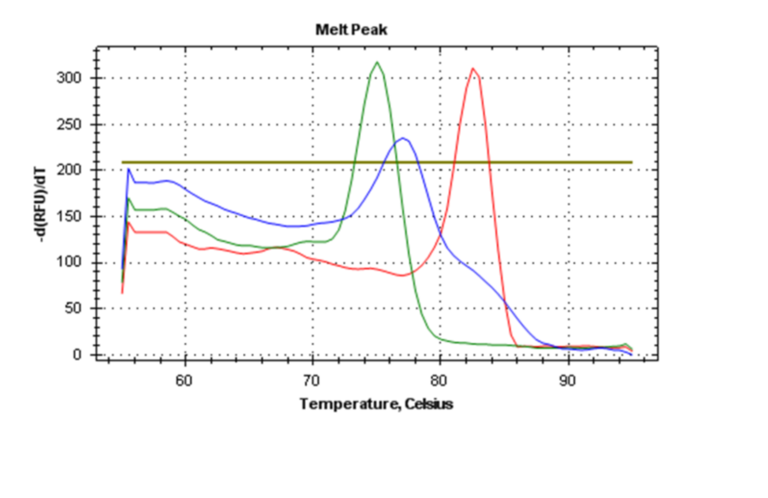

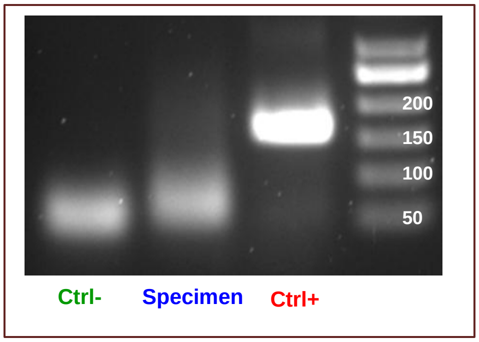

**Analysis**	**Bovine Viral Diarrhea Virus 1, BVDV1**
ID	8727/15-2
Specimen	**Pooled sample of 20 bovine blood specimens**
Method/Gene	Lettelier et al., 2003; *J. Virol. Methods,* **114**, 21-27/ 5'UTR
Amplicon length	168bp
NA extraction	MagNAPure Compact Total NA Isolation Kit (Roche)
qPCR Kit/platform	QuantiTect Probe RT-PCR Kit (Qiagen)/CFX96
Electrophoresis	2% Agarose gel, TAE buffer, 150V

Notes: In this case a TaqMan reaction showed a typical amplification curve of the specimen (blue) in the FAM channel suggests BVDV1 positivity (Cq 30.98). In addition a tail can be observed in the negative control (green).

Surprisingly, this result was not confirmed by the melting analysis. Although, from the first view the FAM channel data was also supported by a curve in the VIC channel the subsequent melting analysis showed a specimen peak (Tm 77°C) that was remarkably shifted away from the positive control (red, Tm difference of 5.5°C). Similarly, the electrophoresis revealed absence of the corresponding specific amplicon.

The MeltMan assay clearly showed that the FAM channel data is false positive. The origin of this false positivity is not known. It apparently resulted from nonspecific probe hydrolysis or disintegration. Such observations are very sporadic and irreproducible. Re-analysis of the identical specimen showed negativity as was suggested by the MeltMan reaction.

Profile no. 3B: Elevated background fluorescence

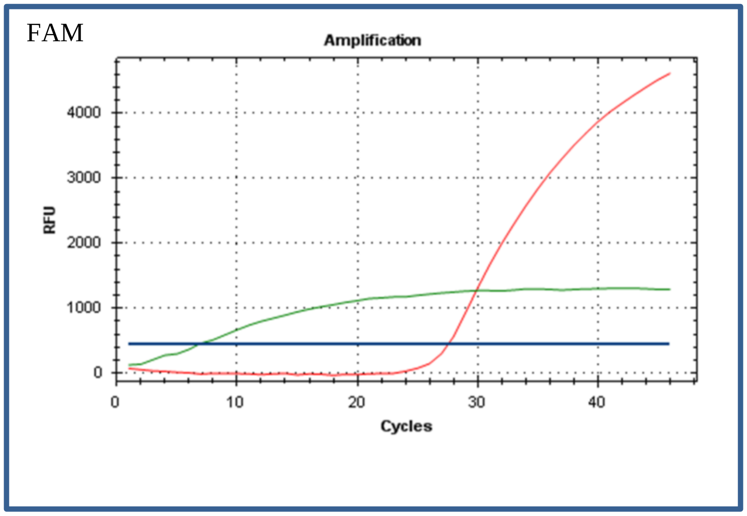

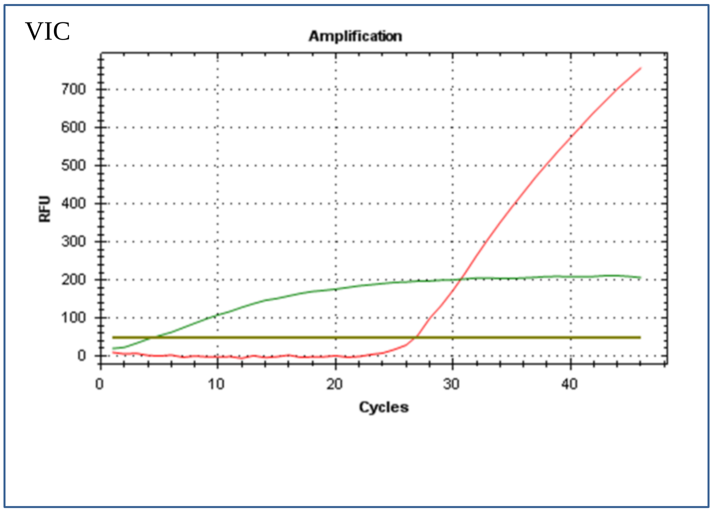

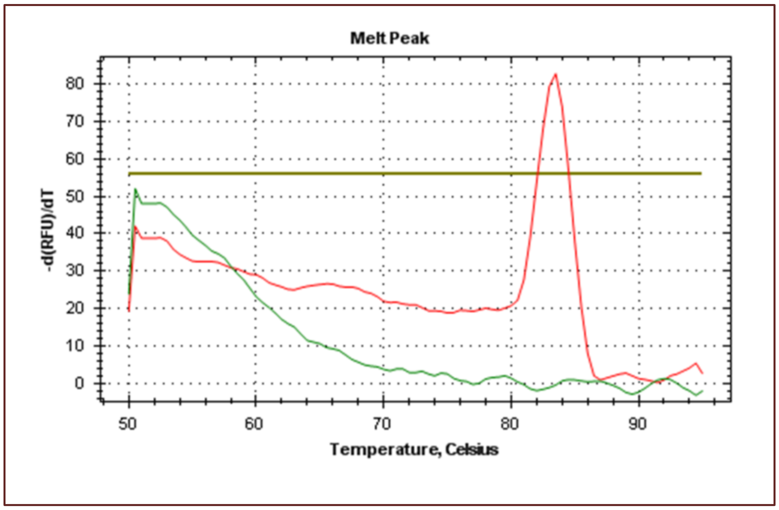

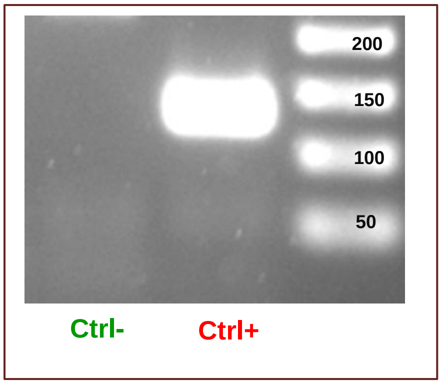

**Analysis**	**n. a.**
ID	n. a.
Specimen	**n. a.**
Method/Gene	**n. a.**
Amplicon length	147
NA extraction	MagNAPure Compact Total NA Isolation Kit (Roche)
qPCR Kit/platform	QuantiTect Probe RT-PCR Kit (Qiagen)/CFX96
Electrophoresis	2% Agarose gel, TAE buffer, 150V

Notes: During the TaqMan reaction a flat curve was observed in the FAM channel. This picture resembles the pattern 1.3, however, in this case the weird curve was generated in the negative control (green). Similar curve trajectory was also observed in the VIC channel.

The MeltMan reaction was able to clearly resolve this observation where the subsequent melting analysis revealed absence of the corresponding amplicon, which was confirmed by electrophoresis. This profile is an example of elevated fluorescence of unknown origin. Such background aberrations can occur in the specimen reactions as well.

Profile no. 4A: Cross species amplification

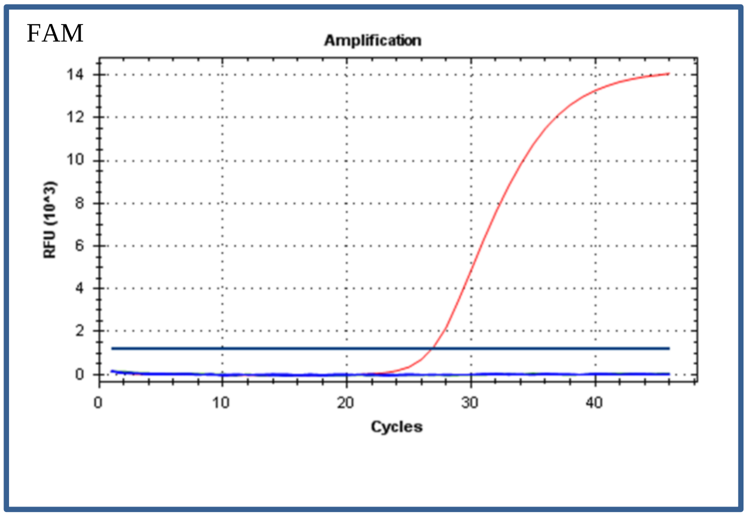

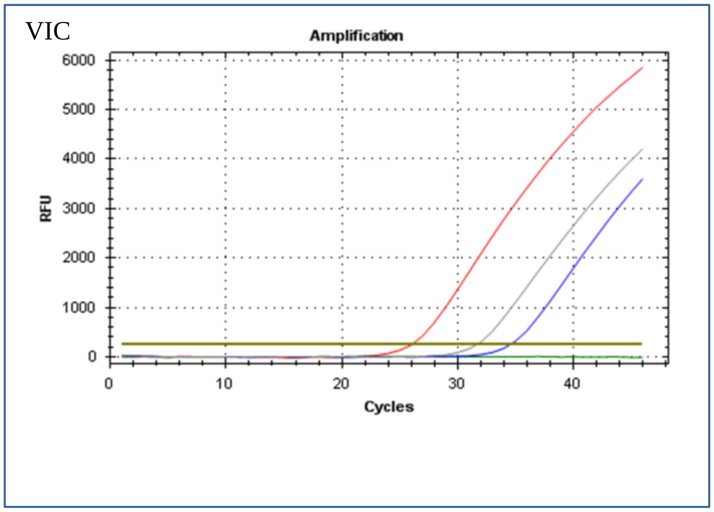

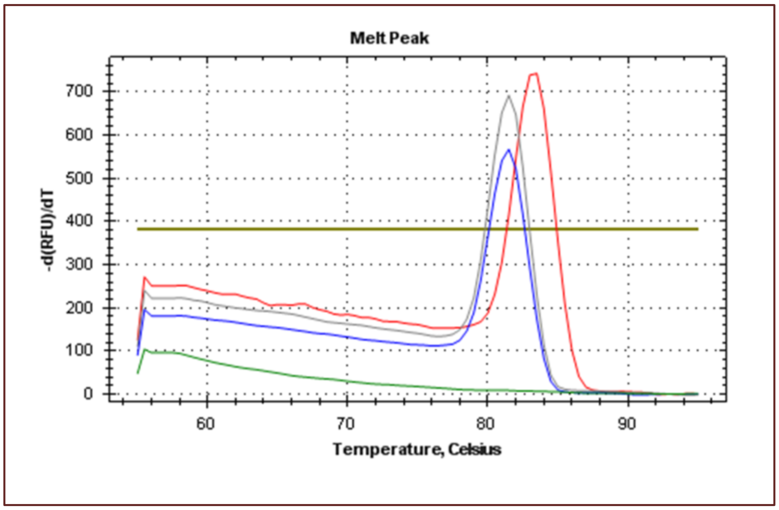

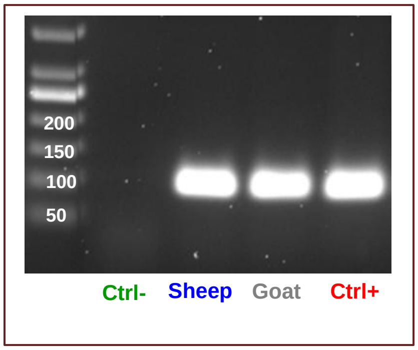


```
                 10        20        30        40        50
        ....|....|....|....|....|....|....|....|....|....|....|.
Bovine  -tgaacctcattctgggg-ccccggcacactcggctgtgttccttgcactcttctg
Sheep   taa.t..c...c.....at....a.......ta..c....................
Goat    taa.t..c...c.....at....a.......ta..c....................
```

**Analysis**	**Bovine DNA**
ID	n.a.
Specimen	**Sheep and goat meat**
Method/Gene	Köppel et al., 2011; *Eur. Food Res. Technol.*, **232**, 151-155/ Beta actin
Amplicon length	88 bp
NA extraction	MagNAPure Compact Total NA Isolation Kit (Roche)
qPCR Kit/platform	QuantiTect Probe PCR Kit (Qiagen)/CFX96
Electrophoresis	2% Agarose gel, TAE buffer, 150V
Sequencing	BDTv3.1, GA 3130 (Life Technologies)

Notes: A bovine DNA specific TaqMan assay was used to analyze a sheep (blue) and goat (grey) meat specimens. The result was bovine DNA negative.

Performing the reaction in MelMan format revealed also amplification in the specimen vials where the melting peak of lower Tm (81.5°C) contrary to the positive control (83.5°C; red) was observed.

Sequencing and BLAST analysis revealed that the TaqMan primers cross reacted with the sheep and goat beta actin genes. So, nonspecific amplification took place in the specimen with a product visible only after the melting analysis. Hence, the TaqMan primers had relaxed template specificity while the bovine DNA specific probe (underlined) was strictly discriminatory. After careful evaluation the cross-amplified products should serve as internal amplification controls.

Profile no. 4B: Cross species amplification

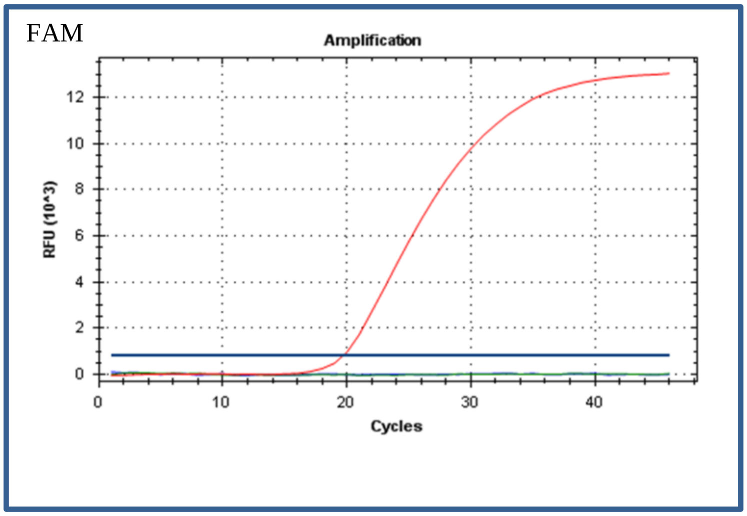

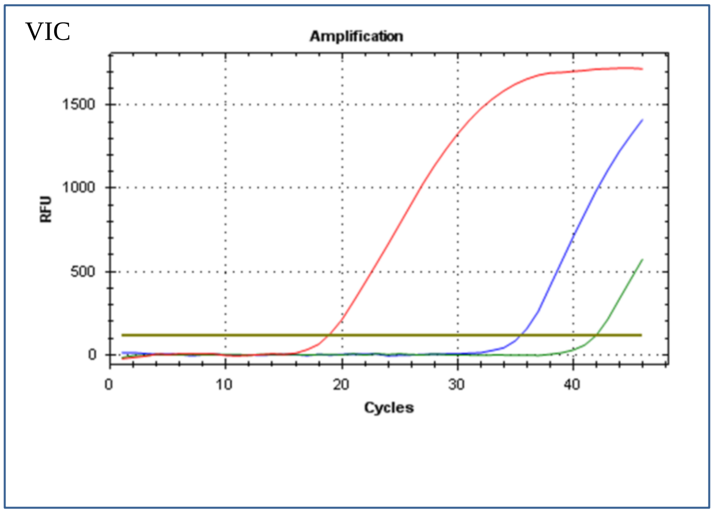

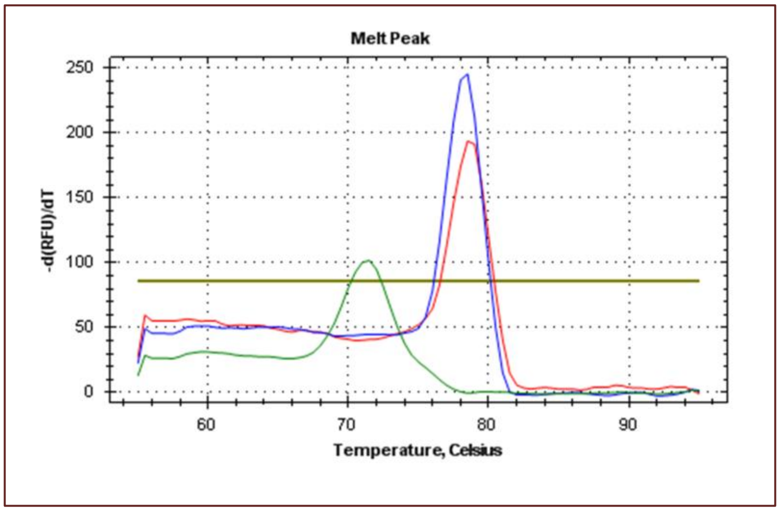

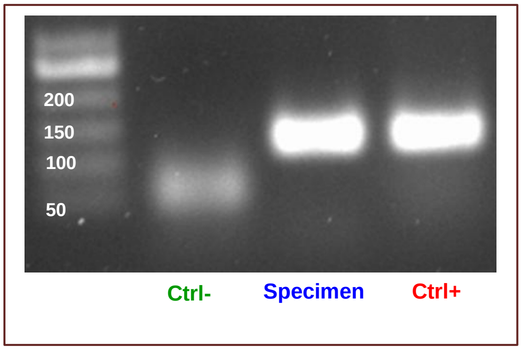

>12657/15
TGAAATAGAACCTGAAACCAGCAGCTTACAAGCGATCAGAGGCCTATGTTC
CTTTTAgGAAAATGGCTGATGGTTTGCCTTTTG

**Analysis**	**Chlamydia sp.**
ID	12657/15
Specimen	**Cat, ocular swab**
Method/Gene	Everett et al., 1999; *J. Clin. Microbiol.*, **37**, 575-580/ 23S rDNA
Amplicon length	129 bp
NA extraction	MagNAPure Compact Total NA Isolation Kit (Roche)
qPCR Kit/platform	QuantiTect Probe PCR Kit (Qiagen)/CFX96
Electrophoresis	2% Agarose gel, TAE buffer, 150V
Sequencing	BDTv3.1, GA 3130 (Life Technologies)

Notes: A chlamydia species specific TaqMan assay was used to analyze a cat ocular swab. The result was negative.

Performing the reaction in MeltMan format also revealed amplification in the specimen vial with identical Tm of 78.5°C.

Sequencing and BLAST analysis showed the closest hit to Fritschea eriococci (GenBank: AY140911) with 90% sequence identity (77/86; 2 gaps) and 100% coverage. This suggests that the 23s rDNA specificTaqMan primers cross reacted with probably a distinct Chlamydia taxon. The obtained demonstrates the universality of the primers used and opens the scope for further in depth studies.

Profile no. 5A: Dye relocation test example

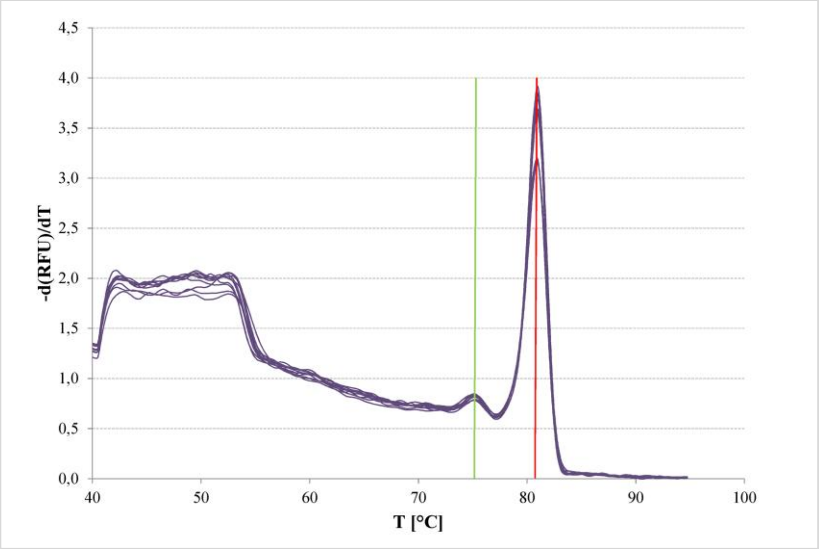

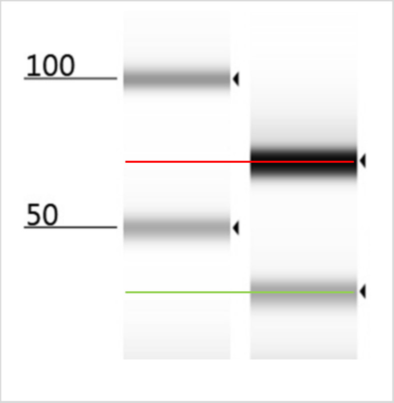

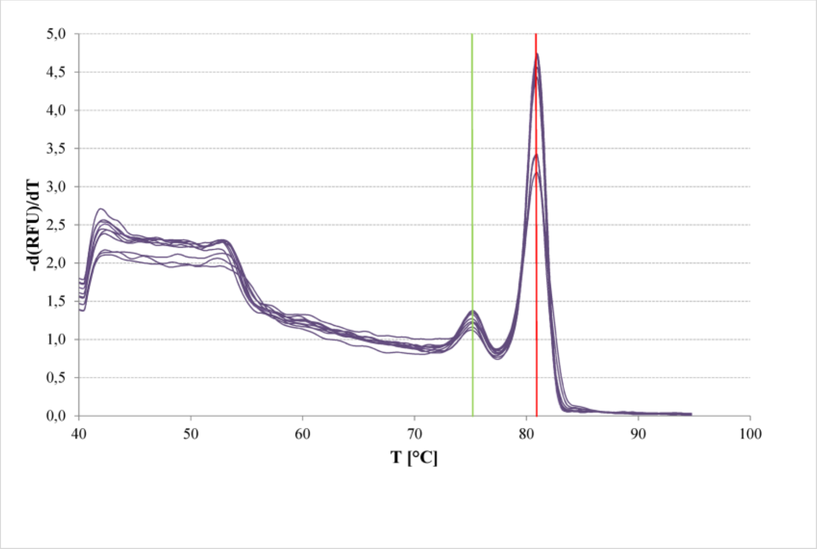

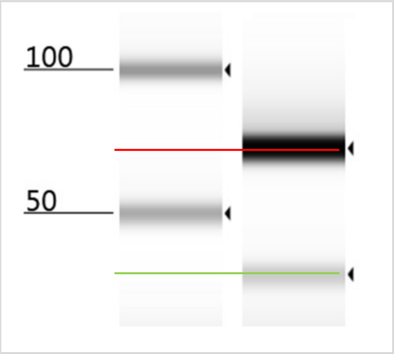

Notes: The dye relocation and melting analysis sensitivity at 0,8μM S82 concentration was tested by ten repeated melting analysis of two specimens showing two different amplicon population sizes (LC480.I). The higher peaks are corresponded with the specific product while the lower peaks are apparently primer dimers. The relative quantity of the specific versus nonspecific amplicons, estimated by the TapeStation 2200, was 11.7: 2.58 and 14.0:1.46 ng/μl respectively. As seen, all the melting replicates gave virtually identical results with no signs of peak disappearing suggesting absence of dye relocation.

Profile no. 5B: Peak discernibility test example

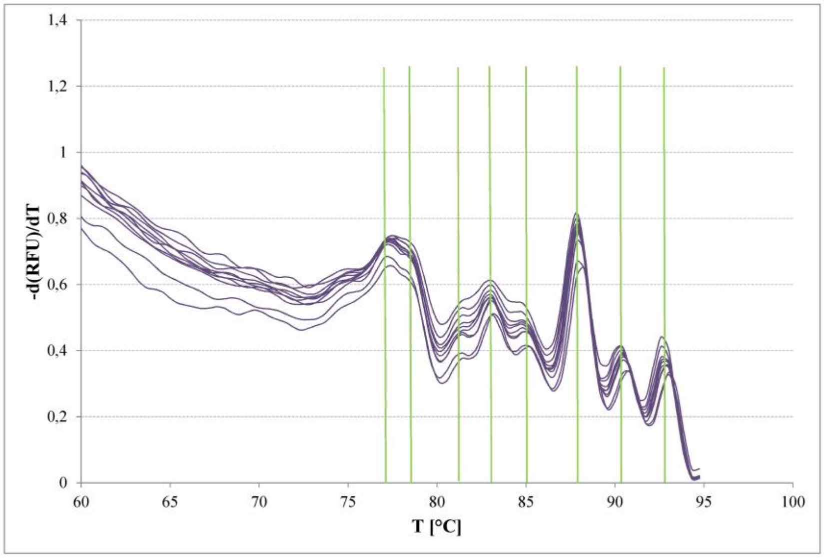

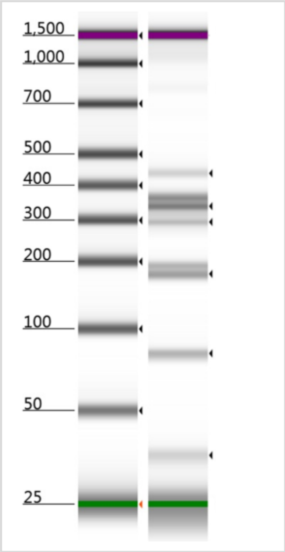

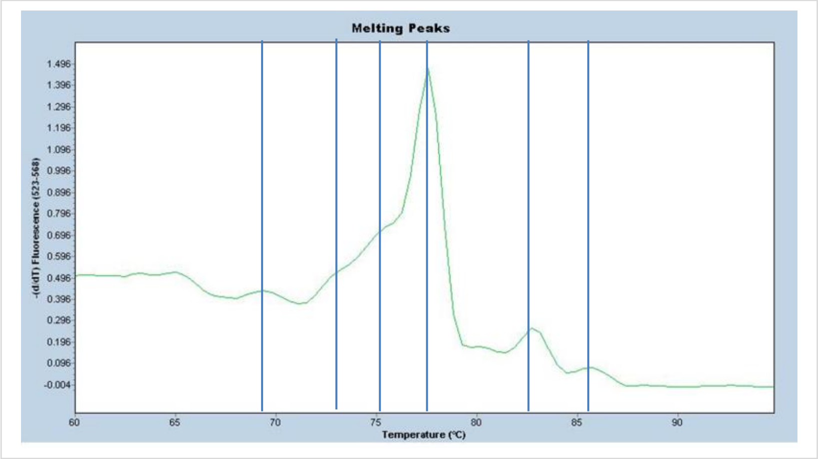

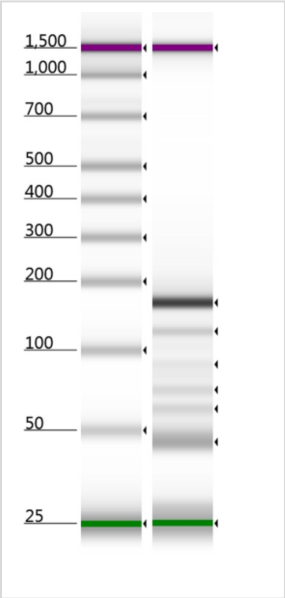

Notes: The discernibility power of the 0.8µM S82 was demonstrated by melting analysis of two RT-qPCR reactions which generated multiple products. The upper figure integrates ten repeated melting analysis of the same reaction while the lower represents a single profile (LC480.I). Although, the discernibility of the melting analysis is greatly influenced by the amplicons Tm values, they abundance, as well as the instrument and melting thermoprofile used the presented example shows correlation between the number of bands in the electrophoreograms and the number of recognisable peaks in the melting profile. However, in the lower figure the peaks with Tm around 72.5 and 75°C tend to coalesce which illustrates the limits of the technique.

Nevertheless, the data proved that the tested S82 concentration sufficient enough for simultaneous visualisation of multiple amplicons, differing in length and abundance, produced in the same reaction. In addition, since all ten replicates in the upper profile showed parallel course the presented example also supports the results of the dye relocation test (Profile 5A).
